# Diet normalization or caloric restriction as a preconception care strategy to improve metabolic health and oocyte quality in obese outbred mice

**DOI:** 10.1186/s12958-021-00848-4

**Published:** 2021-11-04

**Authors:** Anouk Smits, Waleed F. A. Marei, Diane De Neubourg, Jo L. M. R. Leroy

**Affiliations:** 1 Gamete Research Centre, Laboratory for Veterinary Physiology and Biochemistry, Department of Veterinary Sciences, University of Antwerp, 2610 Wilrijk, Belgium; 2 Centre for Reproductive Medicine - Antwerp University Hospital, University of Antwerp, Wilrijkstraat 10, 2650 Edegem, Belgium

**Keywords:** Obesity, Preconception intervention, Diet change, Metabolic health, Oocyte quality, Fertility

## Abstract

**Background:**

Maternal metabolic disorders are linked to reduced metabolic health and oocyte quality. Obese women are advised to lose weight before conception to increase pregnancy chances. However, as human studies show no univocal guidelines, more research is necessary to provide fundamental insights in the consequences of dietary weight loss on oocyte quality. Therefore, we investigated the impact of diet normalization or calorie restricted diet for two, four or six weeks, as preconception care intervention (PCCI), in obese mice on metabolic health and oocyte quality.

**Methods:**

Outbred female mice were fed a control (CTRL) or high-fat (HF) diet for 7 weeks (7w). Afterwards, HF-mice were put on different PCCIs, resulting in four treatment groups: 1) control diet up to 13w, 2) HF diet up to 13w (HF_HF), switch from a HF (7w) to 3) an ad libitum control diet (HF_CTRL) or 4) 30% calorie restricted control diet (HF_CR) for two, four or six weeks. Body weight, metabolic health, oocyte quality and overall fertility results were assessed.

**Results:**

Negative effects of HF diet on metabolic health, oocyte quality and pregnancy rates were confirmed. HF_CTRL mice progressively improved insulin sensitivity, glucose tolerance, serum insulin and cholesterol from PCCI w2 to w4. No further improvements in metabolic health were present at PCCI w6. However, PCCI w6 showed best oocyte quality improvements. Mature oocytes still showed elevated lipid droplet volume and mitochondrial activity but a significant reduction in ROS levels and ROS: active mitochondria ratio compared with HF_HF mice. HF_CR mice restored overall insulin sensitivity and glucose tolerance by PCCI w4. However, serum insulin, cholesterol and ALT remained abnormal. At PCCI w6, glucose tolerance was again reduced. However, only at PCCI w6, oocytes displayed reduced ROS levels and restored mitochondrial activity compared with HF_HF mice. In addition, at PCCI w6, both PCCI groups showed decreased mitochondrial ultrastructural abnormalities compared with the HF_HF group and restored pregnancy rates.

**Conclusions:**

Diet normalization for 4 weeks showed to be the shortest, most promising intervention to improve metabolic health. Most promising improvements in oocyte quality were seen after 6 weeks of intervention in both PCCI groups. This research provides fundamental insights to be considered in developing substantiated preconception guidelines for obese women planning for pregnancy.

**Supplementary Information:**

The online version contains supplementary material available at 10.1186/s12958-021-00848-4.

## Background

The prevalence of obesity and metabolic syndrome is significantly increasing worldwide and has been regarded as a major threat to public health [1–3]. Not only the genetic background but especially a sedentary lifestyle together with a too high caloric intake of diets rich in sugars and (especially) saturated fat are seen as the major causative players [4].

Very often, obesity coincides with a significant reduction in metabolic health, characterized by an increase in body weight, abdominal fat accumulation, aberrant serum lipid profiles, liver dysfunction, an impaired glucose tolerance and reduced insulin sensitivity [5–7]. Reduced fertility is often seen in these obese patients. Clinical centers for assisted reproduction report a higher incidence of menstrual irregularity or even anovulation, reduced oocyte developmental capacity after in vitro fertilization and thus a longer time to successful conception in this cohort of obese patients [8–10]. Even obese women with a normal ovarian cycle display reduced fertility rates, indicating a negative impact of the disturbed metabolic health on critical peri-conception events that determine oocyte quality and ultimate pregnancy success [11].

In depth research, using not only human but also animal research models, provide growing evidence for the major role that reduced oocyte quality plays in the pathogenesis of subfertility in metabolically compromised women. We and others showed that the oocyte’s micro-environment, the follicular fluid (FF), in obese women reflects the disturbed metabolic state [6, 12]. This means that the oocyte directly senses the hyperglycemic and hyperinsulinemic conditions [6] next to the elevated non-esterified fatty acid (NEFA) and triglyceride concentrations [13]. Factors involved in oxidative stress and inflammation also impact on the FF composition and have the potential to reduce oocyte quality [14, 15].

Oocytes, collected from obese patients but also from Western Type diet induced obese mice, displayed an impaired quality, indicated by high rates of meiotic spindle abnormalities, increased mitochondrial ultrastructural abnormalities, altered mitochondrial membrane potential and increased cellular oxidative stress levels [16–19]. Furthermore, mitochondrial DNA (mtDNA) copy number and mitochondrial biogenesis in oocytes from obese mice were altered [18] and higher levels of lipid accumulation and abnormal lipid distribution in oocytes were present [18–20].

Up until now, overweight and obese patients are often advised to lose weight before conception through dietary lifestyle interventions to improve metabolic health, fertility and to increase the chance of a healthy pregnancy [6, 21, 22].

Although a limited amount of weight loss (3–5%) can already partly improve metabolic health in humans, more significant weight losses are needed for complete recovery [23]. Such a weight loss and metabolic health improvement can be achieved by a simple diet normalization [20]. However, in a lot of studies, a more strict calorie restricted diet showed to be more effective for weight loss and restoring metabolic health [24, 25]. Extreme weight loss induces extensive lipid mobilization, and thus high NEFA concentrations, which may trigger lipotoxic effects in the oocyte [26]. In depth in vitro research could clearly confirm the importance of these lipotoxicity pathways in explaining the reduced oocyte quality seen in metabolically compromised individuals [27]. Such direct impact of drastic weight loss regimes on oocyte quality has, to the best of our knowledge, never been studied before.

So far, there are no evidence-based guidelines regarding fertility treatment in overweight and obese infertile women. Several (sometimes underpowered) lifestyle intervention studies that investigated the effect of weight loss before conception on fertility in obese and overweight individuals, observed a significant increase in pregnancy and/or live birth rates (for an overview, see [28]). Recent large randomized controlled trials could not confirm this [29, 30] and concluded that preconception weight loss via dietary interventions did not improve live birth rates in obese women scheduled for IVF [29]. However, the authors did detect more spontaneous pregnancies in the lifestyle program group. Advising for a more severe weight loss before conception by applying a very low calorie diet, has recently been discouraged in clinical settings as it is estimated that this results in potential harm to the oocyte which may further lead to an adverse pregnancy outcome [23, 31].

These conflicting results, together with the limitations of human studies (high drop-out rates, lack of sufficient power, patient clinical history, societal and lifestyle background) lead to a lack of scientifically supported advice [32]. Does significant weight loss, as a preconception care intervention, have a positive impact on oocyte quality and/or might diet normalization be sufficient to optimize fertility outcome? Only very few studies focused on the impact of preconception dietary interventions on oocyte quality in obese women, so in depth research is needed.

Reynolds et al. used an inbred obese mouse model (C57BL/6) for her research and reported that diet normalization for 8 weeks could not recover oocyte quality although the metabolic health returned to normal. However, we recently showed that control C57BL/6 mice, in contrast to outbred Swiss mice, already show a high degree of oocyte mitochondrial abnormalities and a disturbed mitophagy [18]. Therefore, using an outbred mouse strain might be more relevant for this research and for further translation to human settings. A small pilot study reported a negative outcome on in vitro fertilization rates after feeding obese women a short term very low calorie diet for 4–6 weeks [31]. It is not known if a longer exposure time might be more efficient to improve oocyte quality or whether a long-lasting carry-over effect at the early phase of folliculogenesis should be expected on the quality of the mature oocyte.

This confirms the need for well-controlled and strategically designed outbred animal experiments investigating the specific effect of diet normalization or caloric restriction and the duration of these interventions on oocyte quality. Are a complete normalized weight and metabolic health necessary for oocyte quality to recover? It is important to emphasize that this intervention period should be as short as possible to avoid the potential negative effects of advancing maternal age on ovarian reserve and reproductive capacity [28, 33].

Therefore, in this study we hypothesized that the efficiency of a preconception care intervention (PCCI) in high fat-fed obese outbred mice to improve metabolic health, oocyte quality and fertility depends on the method of diet change (diet normalization or caloric restriction) and on the duration of that intervention.

## Aims of the study

To test this hypothesis, we aimed to switch high fat-fed obese outbred mice to two different preconception care interventions (PCCIs): 1) an ad libitum control diet or 2) a 30% calorie restricted control diet for two, four or six weeks and to assess the impact on metabolic health, oocyte quality and general fertility results.

To assess the impact on metabolic health we aimed to analyze serum insulin, glucose, cholesterol, triglyceride, NEFA and alanine aminotransferase (ALT) concentrations together with the assessment of glucose tolerance and insulin sensitivity (at PCCI week 0, 2, 4 and 6). Oocyte quality was evaluated by assessing intracellular lipid droplet content, reactive oxygen species (ROS), mitochondrial activity and localization of active mitochondria, as well as mitochondrial ultrastructural abnormalities, and mtDNA copy numbers. In addition, oocyte recovery and pregnancy rates were investigated.

## Materials and methods

### Animals, diet and experimental design

Five-week-old female outbred Rj:Orl Swiss (*n* = 156, hereafter referred to as “Swiss”) mice (Janvier labs) were used. At the start of the experiment, mice were randomly divided into two groups with ad libitum access to either a control (CTRL, E157453-04; Sniff Diets) or a high fat diet (HF, E15741-34, Sniff diets) for a period of 7 weeks.

**Fig. 1:**
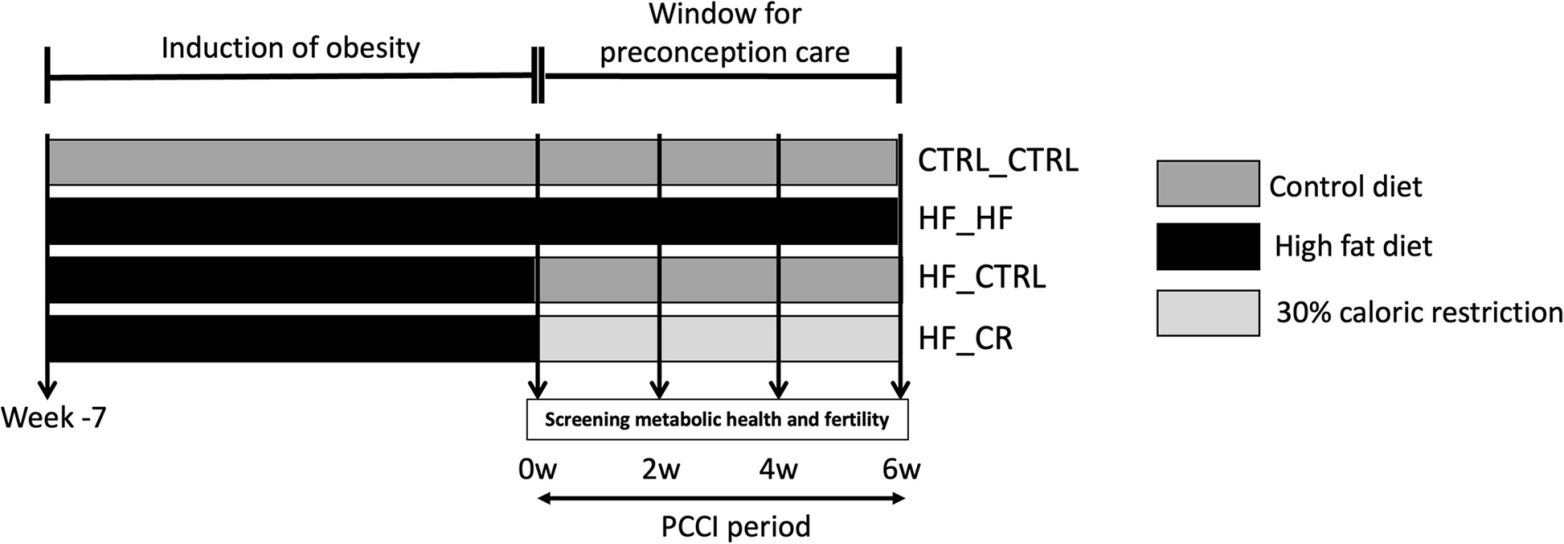
Overview of the experimental design

Afterwards, some of the HF-mice were switched to two different preconception care interventions for two, four or six weeks, while the remaining HF and the control mice remained on their corresponding diet for comparison. This resulted in four different treatment groups of equal size: 1) control diet for up to 13 weeks (CTRL_CTRL), 2) high fat diet for up to 13 weeks (HF_HF), 3) high fat diet for 7 weeks then a switch to an ad libitum control diet for two, four or six weeks (HF_CTRL) and 4) high fat diet for 7 weeks then a switch to a 30% caloric restriction diet for two, four or six weeks (HF_CR). An experimental timeline is shown in Fig. 1.

The HF diet was composed of 60 kJ% fat from beef tallow, 20 kJ% carbohydrate and 20 kJ% protein. The matched, purified control diet contained 10 kJ% fat, 70 kJ% carbohydrate and 20 kJ% protein. Access to water was provided ad libitum. The 30% caloric restriction diet had the same composition as the control diet, however, the HF_CR mice received only 70% of the amount of food consumed by the CTRL_CTRL mice (paired feeding regimen, calculated on the basis of the daily consumption).

Mice were put on the diet in subgroups of 8 animals per treatment per time point (in 2 replicates) with an interval of few weeks between replicates to facilitate handling and sample collection procedures. Mice were weighted weekly before the start of the PCCI and every 4 days during the PCCI period to closely follow-up weight changes.

### Serum collection and analysis

At each time point during the PCCI period, mice were fasted overnight and were sacrificed by decapitation to allow blood collection. Blood was centrifuged 30 min after collection at 2000 rpm for 10 min, and serum was stored at − 80 °C until analysis. Insulin concentrations were measured using an ultrasensitive mouse insulin ELISA kit (90,080, CrystalChem, The Netherlands). In addition, the following serum analyses were performed in a commercial laboratory (Algemeen Medisch Labo, Antwerp, Belgium): NEFA concentrations were determined using a colorimetric assay (Randox Laboratories Ltd., Crumlin, Co. Antrim, United Kingdom) on an IDS iSYS multi-discipline automated instrument (Immunodiagnostic Systems Hld, Tyne & Wear, UK); Triglycerides, Cholesterol and Alanine aminotransferase (ALT, an overall indicator of liver damage), were measured on an Abbott Architect c16000 (Abbott, Illinois, U.S.A).

### Insulin and glucose tolerance test

Mice (*n* = 8 per treatment per time point) were fasted for 6 h prior to the glucose (GTT) and insulin tolerance test (ITT). The tip of the tail was cut off and a blood drop was used to measure the basal glycaemia with a glucose stick (One Touch Verio glucose test strips; ADC, Belgium) in a glucose meter (OneTouch Verio; ADC, Belgium) and immediately afterwards the mouse was intraperitoneally injected with 20% glucose solution (2 g glucose/kg body weight; Thermo Fisher Scientific, Belgium) for the GTT or with 0,075 IU insulin/kg body weight (Novorapid (Novo Nordisk), ADC, Belgium) for the ITT. The glycaemia was measured again 15, 30, 60, 90 and 120 min after the injection. Afterwards, the area under the curve (AUC) and elimination rate (ER) of glucose were calculated for both tests as follows:

$$AUC=\left(\left({C}_1+{C}_2\right)/2\right)\times \left({t}_2-{t}_1\right)$$

C1 and C2 are the concentrations of glucose at time points t1 and t2, respectively [34]. This calculation was made per time frame (0-15 min, 15-30 min etc.) and total AUC was calculated as the sum of all AUC calculations.

$$\mathrm{ER}\;\mathrm{gluc}=\left\{\left(\ln \left[\mathrm{P}\;\mathrm{gluc}\right]\hbox{-} \ln \left[\mathrm{N}\;\mathrm{gluc}\right]\right)/\left(\mathrm{tp}\hbox{-} \mathrm{tn}\right)\times 100\right\}$$

*Pgluc* and *Ngluc* are peak and nadir (lowest) glucose concentrations, while tp and tn are the times of the peak and nadir glucose concentrations, respectively [35].

The same mice were used to perform GTT and ITT with an interval of 2 days. The analysis was repeated using the same mice at zero, two, four and six weeks of PCCI (*n* = 8 per treatment group).

### Oocyte and cumulus cell collection and preparation for subsequent analyses

In order to avoid any bias due to the glucose and insulin injections, different mice than those used for the GTT and ITT were selected to determine oocyte quality. At all time points, these mice received intraperitoneal injections of 10 IU equine chorionic gonadotropin (eCG, Synchrostim; Ceva Santé Animale) followed, 48 h later, by 10 IU human chorionic gonadotropin (hCG, Pregnyl; Organon) to induce and synchronize ovulations. Mice were sacrificed 13–14 h after hCG injection. In vivo matured oocytes were obtained from the oviducts immediately after euthanasia. Each oviduct was dissected together with the ovary and a part of the uterine horn and transferred to a collection tube containing L15 medium (Thermo Fisher Scientific, Belgium) supplemented with 50 IU/mL penicillin G sodium salt (Merck, Belgium), and 10% Fetal Bovine Serum (Greiner Bio-One, Belgium).

The cumulus oocyte complexes (COCs) collected from both oviducts of the same animal were pooled. Only COCs that met the following selection criteria were used for analysis: oocytes surrounded by an expanded cumulus cell mass with a perfect spherical shape, a regular zona and a translucent, homogeneously colored cytoplasm without inclusions. Morphologically good quality COCs with expanded cumulus cells from each mouse were distributed for downstream analysis, according to the total number of COCs available, as follows: one whole COC per mouse was fixed in glutaraldehyde solution for transmission electron microscopy (TEM) (at 0 and 6w of PCCI). The remaining COCs were completely denuded by repeated pipetting through 150 μm Stripper tips fitted on EZ-grip (Origio, The Netherlands) in a droplet of L15 medium supplemented with 0.3 mg/mL hyaluronidase (Merck, Belgium). Denuded oocytes were transferred to a fresh drop of L15 medium. One or two denuded oocytes (per mouse) were immediately transferred to JC-1 and CellROX Deep Red staining to determine mitochondrial activity and intracellular ROS content. One to two oocytes per mouse were fixed in paraformaldehyde 4% for determination of lipid droplet content. The remaining oocytes were washed in PBS containing 1 mg/ mL PVP and snap frozen per individual mouse in a 1.5 mL tube in a minimum volume for DNA extraction for determination of mtDNA content. All frozen samples were stored at − 80 °C until further analyses.

An overview of all outcome parameters at specific time points (week 0, 2, 4 and/or 6 after start of the PCCI) are presented in Table 1. The final numbers of animals and oocytes used to collect data for each outcome parameter are described in the figure legends.

**Table 1:** Overview of the outcome parameters assessed at specific time points during the preconception care intervention period (PCCI)

		PCCI week 0	PCCI week 2	PCCI week 4	PCCI week 6
Metabolic health	Insulin sensitivity test^a^ (*n* = 8 mice/treatment group/time point)	✓	✓	✓	✓
	Glucose tolerance test^a^ (*n* = 8 mice/treatment group/time point)	✓	✓	✓	✓
	Serum^b^ - Glucose - Insulin - NEFA - Cholesterol - Triglycerides - ALT (*n* = 7–8 mice/treatment group/time point)	✓	✓	✓	✓
Fertility	Oocyte recovery rate^b^ (*n* = 8 mice/treatment group/time point)	✓	✓	✓	✓
	Lipid droplet volume in oocytes^b^ (*n* = 8 mice/treatment group/time point)	✓	✓	✓	✓
	ROS in oocytes^b^ (*n* = 8 mice/treatment group/time point)	✓	✓	✓	✓
	Mitochondrial activity in oocytes^b^ (*n* = 8 mice/treatment group/time point)	✓	✓	✓	✓
	Localization of active mitochondria in mature oocytes^b^ (*n* = 8 mice/treatment group/time point)	✓	✓	✓	✓
	mtDNA copy numbers in oocytes^b^ (*n* = 3–5 mice/treatment group/time point)		✓	✓	✓
	Mitochondrial ultrastructural abnormalities (TEM)^b^ in cumulus cells and oocytes (*n* = 3–5 mice/treatment group/time point)	✓			✓
	Pregnancy rates (*n* = 8 mice/treatment group)				✓

*NEFA* non-esterified fatty acids, *ALT* alanine aminotransferase, *ROS* reactive oxygen species, *TEM* transmission electron microscopy

^a^Same mice were used to determine the selected outcome parameters over all PCCI time points

^b^ Same mice were used per PCCI to analyze the selected outcome parameters

### Assessment of oocyte lipid droplet volume

Intracellular lipid droplets in the fixed denuded oocytes were examined using BODIPY staining according to Marei WFA*,* et al. [18]. To summarize, oocytes were permeabilized for 30 min in PBS containing 0.1% (w/v) saponin (Fiers, Kuurne, Belgium) and 0.1 M glycine. Next, oocytes were incubated in 20 μg/ml BODIPY 493/503 (Thermo Fisher Scientific, Belgium) in PBS for 1 h. Oocytes were washed twice in PBS containing 3 mg/mL PVP after each step in the staining procedure. Finally, the oocytes were transferred to droplets of PBS-PVP on glass-bottom dishes and immediately examined under a confocal microscope. High resolution images were obtained using a Nikon Eclipse Ti-E inverted microscope attached to a microlens-enhanced dual spinning disk confocal system (UltraVIEW VoX; PerkinElmer, Zaventem, Belgium) equipped with 488 nm diode lasers for excitation of green fluorophores, respectively. For each oocyte, a z-stack of 40 μm (with steps of 1 μm) was acquired in the lower half of the oocyte (closest to the objective lens where the image is sharpest). Images were analyzed using Volocity 6.0.1 software (PerkinElmer) to evaluate the differences in lipid droplet content among oocytes in different groups. To exclude background, only particles ≥0.5 μm^3^ in size were considered as lipid droplets and included in the analysis.

### Assessment of mitochondrial activity, localization of active mitochondria and intracellular ROS

Oocyte mitochondrial activity and intracellular ROS concentrations were assessed using a combined fluorescence staining technique using 5,5′,6,6′-tetrachloro-1,1′,3,3′-tetraethylbenzimidazolyl-carbocyanine iodide (JC-1, Invitrogen) and CellROX™ Deep Red Reagent (Thermo Fisher Scientific, Belgium) as described by Komatsu K*,* et al. [36]. This combined staining and simultaneous detection using multilaser was validated and described by De Biasi S*,* et al. [37]. Freshly collected oocytes were incubated for 30 min in L15 medium containing JC1 (5 μg/mL) and CellRox deep red (2.5 mM) (from 1000X stock solutions in DMSO) at 6% CO_2_ and 37 °C. They were then washed and transferred to L15 medium droplets under mineral oil on a 35 mm dish with a glass bottom. Stained oocytes were immediately examined under a Leica SP8 confocal microscope enclosed in a humid warm chamber (37 °C) and equipped with white laser source (Leica WLL) lasers. Mitochondrial activity was measured as the mean grey scale intensity of the J-aggregates (excitation/emission 561/590 nm, which is dependent on mitochondrial inner membrane potential), while intracellular ROS was measured as the mean grey scale intensity of the Cell Rox deep Red (644/665 nm). The ratio of ROS: active mitochondria was calculated as the ratio of grey scale intensity at 665:590. The grey scale intensity in each channel was measured using Leica Application Suite X (LAS X) software.

The regional distribution of active mitochondria was examined. Active mitochondria migrate within the developing oocyte. They reach a pericortical localization in the mature oocyte, which is linked to successful preimplantation development [38]. To determine pericortical localization of active mitochondria, one optical section was taken (excitation/emission 561/590 nm) at the maximum oocyte diameter. Based on its radius, the oocyte was divided into 5 equal circular zones [38], in which the most central zone was labelled as zone 1 and the most peripheral zone was labelled as zone 5. If mitochondria were localized in zone 5 of the oocyte, they were categorized as “Pericortical”. If not, they were labelled as “Diffuse”. Since this staining is performed on live oocytes, the number of oocytes/mouse used in this outcome parameter was limited to avoid any bias due to increased time of imaging.

### Mitochondrial ultrastructure - transmission electron microscopy (TEM)

The ultrastructure of mitochondria in cumulus-oocyte-complexes was only assessed at zero and 6 weeks of PCCI, according to Marei WFA*,* et al. [18]. Briefly, freshly collected whole COCs were immediately fixed in 0.1 M sodium cacodylate-buffered (pH 7.4) 2.5% glutaraldehyde solution at 4 °C for a maximum of 1 month. Individual COCs were then embedded in 2% agarose blocks to enable handling. Afterwards, blocks were washed three times in 0.1 M sodium cacodylate-buffered (pH 7.4) 7.5% saccharose solution. Post-fixation was performed by incubating the blocks for 2 h with 1% OsO4 solution. After dehydration in an ethanol gradient, samples were embedded in EM-bed812. Ultrathin sections were stained with lead citrate, and examined in a Tecnai G2 Spirit Bio TWIN microscope (Fei, Europe BV, Zaventem, Belgium) at 120 kV. For each COC, images of at least 5 cumulus cells and at least 10 random fields in the oocyte (covering most of the oocyte area), were acquired at 16500–25000×. Mitochondria in the acquired images were morphologically evaluated by an expert blind to the corresponding treatment group and were classified based on their morphology (Supplementary Fig. 1), according to Marei WFA*,* et al. [18].

### Relative change of mtDNA copy numbers

DNA extracts from oocyte pools were used to determine the ratio of mtDNA to nuclear DNA by qPCR of the mitochondrial gene (ND4) and the nuclear gene (bACT). The relative mtDNA: nuclear DNA ratio was calculated using the 2–ΔΔCq method described by Livak and Schmittgen [39].

### Pregnancy rates

As an endpoint assessment (PCCI week 6), mice (*n* = 8 from each treatment group) were mated with Swiss males of proven fertility (maintained on a control diet) to test for pregnancy rates. Two females were housed with one male for four nights. During mating and pregnancy, CTRL_CTRL, HF_HF and HF_CTRL mice stayed on their respective diet. HF_CR mice were offered an ad libitum control diet to provide sufficient nutrients to the fetuses during pregnancy. Pregnancy rates were assessed between 18 and 21 days after mating.

### Statistical analysis

Statistical analysis was performed with IBM SPSS Statistics 26 (for Windows, Chicago, IL, USA).

Numerical data, e.g. weight, blood parameters, mitochondrial activity and ROS, were checked for normal distribution and homogeneity of variance. Within each time point, numerical data were analyzed using One-way ANOVA. Post-hoc LSD was performed in a sequential manner for predefined comparisons based on the null hypothesis for each conditional research question. Following research questions were covered: 1) did exposure to a HF_HF diet induce a change compared with the CTRL_CTRL, if yes: 2) where the PCCIs effective in achieving any IMPROVEMENT compared with the HF_HF group, if yes: 3) were the measurements in the PCCI groups RECOVERED to the level of the CTRL_CTRL group. To test questions 1 and 2, a post-hoc test was performed using the HF_HF group as a reference group. If a significant difference (and thus an improvement) was detected in the PCCI group(s) compared with the HF_HF group, a second post-hoc test was performed using the CTRL_CTRL group as a reference group to check for potential recovery (i.e. considered as no significant difference anymore between a PCCI group and the CTRL_CTRL group). Non-homogenous data were analyzed using non-parametric independent sample Kruskal Wallis and a series of Mann-Whitney t-tests using the same sequential approach. On the other hand, categorical data, e.g. proportions of different ultrastructural classifications in TEM images, were analyzed using a Chi Square test also using the same strategy for comparisons.

The number of mice and oocytes used to generate the data are described in the results section for each parameter. Differences with *P-*values ≤0.05 were considered statistically significant. Differences with *P* values> 0.05 and ≤ 0.1 were reported as tendencies. Data are expressed as means ± S.E.M unless otherwise stated.

### Sample size calculation

Sample size calculation was performed using ‘PS: Power and Sample Size Calculation version 3.1.2, 2014 (from Vanderbilt University)’. Based on available data from relevant outcome parameters (serum concentrations, glucose tolerance test, staining for oocyte lipid droplet volume), numbers of mice needed to detect statistical differences, averaged between 7 and 8. Sample size calculation was performed with a type 1 error of 0.05 and a power of 0.9.

## Results

### Weight gain and loss

**Fig. 2:**
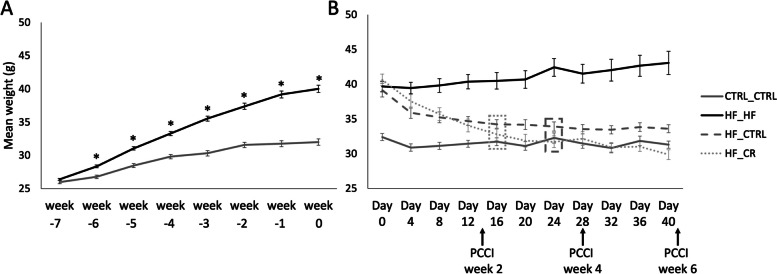
The effect of high fat diet on body weight gain during the first 7 weeks (**A**); and body weight changes after the start of the preconception care intervention (PCCI) period (**B**). Data of (**A**) are shown as means ± SEM from 156 mice in total. Significant difference (*P* < 0.05) between HF_HF and CTRL_CTRL group are indicated by an asterisk (*). Time point at which HF_CR or HF_CTRL group showed no significant difference anymore with the CTRL_CTRL group are indicated by a rectangle with the same lay-out (**B**)

Feeding a HF diet already resulted in a significantly higher weight in HF_HF mice after only 1 week when compared with mice fed the control diet (CTRL_CTRL). HF-fed mice kept increasing in weight resulting in 25% more weight than the control group after 7 weeks on the HF-diet (Fig. 2a). As soon as the PCCI period started, mice that switched from a HF to an ad libitum control (HF_CTRL) or a 30% caloric restricted control (CR) diet (HF_CR) started to lose weight (Fig. 2b). After 16 days of PCCI, the HF_CR group showed a mean weight loss of 20.04%, and reached similar weights as the CTRL_CTRL group. As the PCCI continued, HF_CR mice kept losing weight however never significantly below the weight of the CTRL_CTRL mice. In the HF_CTRL group, complete weight recovery was achieved after 24 days of PCCI with a weight loss of 13.34%.

### Metabolic health

#### Blood serum profile

**Fig. 3:**
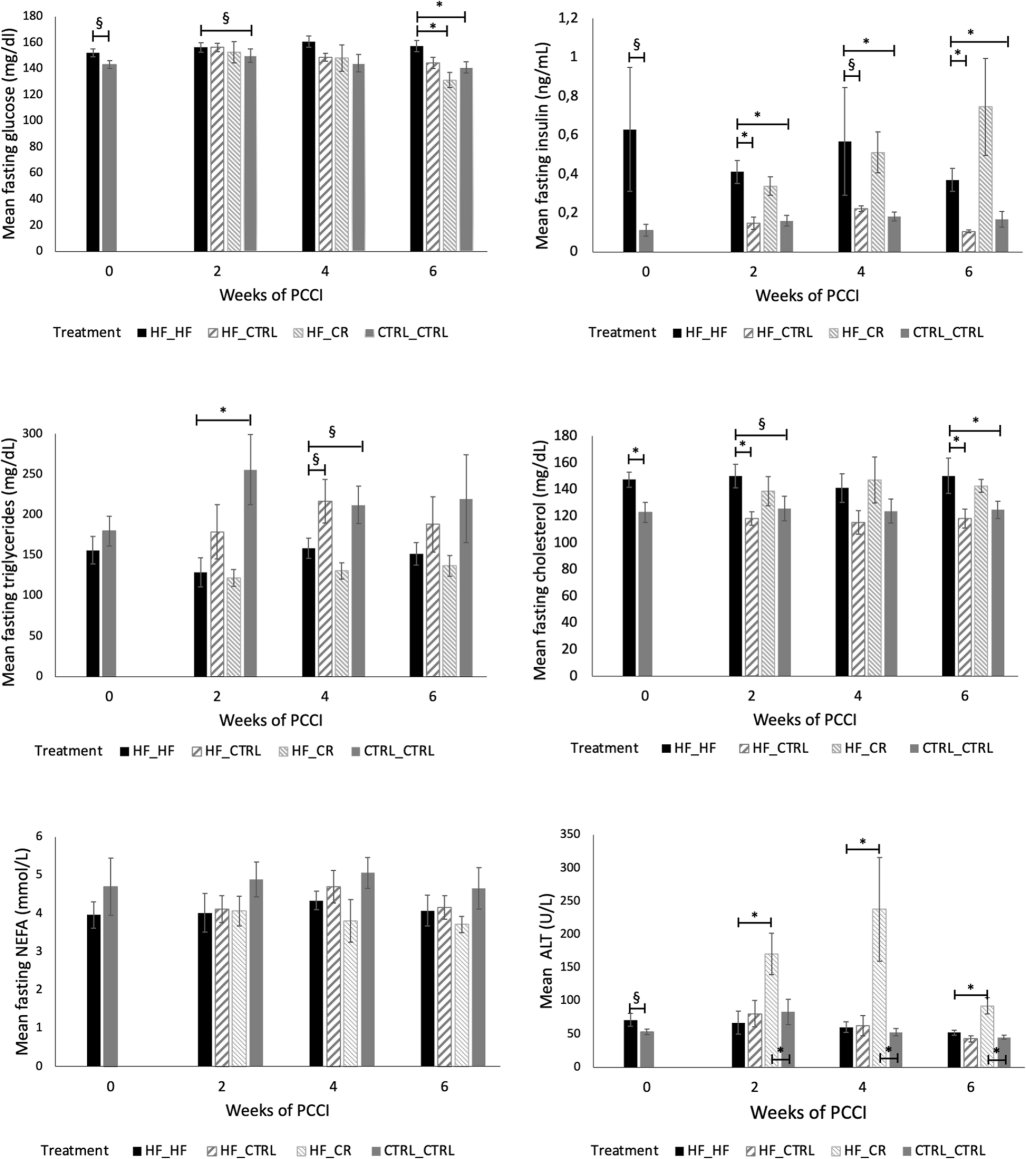
Fasting serum concentrations of glucose, insulin, cholesterol, triglycerides, non-esterified fatty acids (NEFA), and alanine aminotransferase (ALT) among all treatment groups at different time points after starting the preconception care intervention (PCCI). Data are shown as means ± SEM from 7 to 8 mice per group per time point. Insulin concentrations after zero weeks of PCCI are from 4 mice/treatment group. Asterisks (*) indicate significant differences between the indicated treatment groups within the same PCCI period (*P* < 0.05). Values labelled with “§” tend to be different from each other at 0.05 < *P* < 0.1

Feeding a HF diet increased fasting blood glucose concentration compared with the CTRL_CTRL group after zero (*P* < 0.1), two (*P* < 0.1) and six (*P* < 0.05) weeks of PCCI (Fig. 3). This was associated with a marked increase in serum fasting insulin concentrations at all time points. Switching from a high fat to an ad libitum control diet (HF_CTRL) did not improve serum glucose concentrations when compared with the HF_HF group. However, serum insulin concentrations were normalized starting from week 2 (*P* < 0.001 at week 2, *P* < 0.1 at week 4 and *P* < 0.001 at week 6).

In contrast, insulin levels in the HF_CR group remained as high as in the HF_HF mice at all time points (*P* > 0.1) while these mice only displayed significantly improved glucose concentrations after 6 weeks of PCCI (*P* < 0.05) when compared with the HF_HF group.

When focusing on the blood lipid profile, HF_HF mice displayed higher fasting cholesterol concentrations when compared with the CTRL_CTRL group at almost all time points (PCCI week 0 (*P* < 0.05), week 2 (*P* < 0.1) and week 6 (*P* < 0.05); Fig. 3). Mice that underwent diet normalization (HF_CTRL) already showed restored cholesterol concentrations after only 2 weeks of PCCI (*P* < 0.05). In contrast, HF mice submitted to the CR diet (HF_CR) never showed any improvement of the elevated cholesterol concentrations. No significant differences in fasting NEFA concentrations could be detected in our experimental set-up.

Interestingly, triglyceride concentrations in the HF_HF group were lower after two (*P* < 0.05) and four (*P* < 0.1) weeks of PCCI when compared with the CTRL_CTRL group. Feeding a control diet to obese mice as a PCCI (HF_CTRL) only tended to increase triglyceride concentrations again after 4 weeks of intervention (*P* < 0.1). A 30% calorie restricted diet (HF_CR) had no impact at all on the lowered triglyceride concentrations seen in the HF_HF group.

Feeding a HF diet had no effect on ALT concentrations, which is an indicator of liver damage. HF_CR mice, however, showed significantly higher ALT concentrations than the control (CTRL_CTRL) and the high fat (HF_HF) group at all time points.

#### Insulin and glucose tolerance tests

Glucose tolerance tests (GTT) were performed at all time points on the same mice per treatment group.

**Fig. 4:**
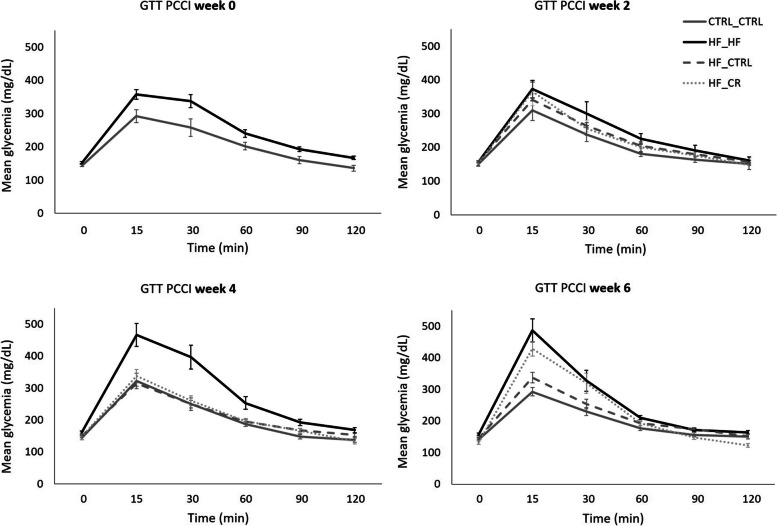
Serum glucose concentrations during a glucose tolerance test (GTT) per treatment group at different time points after starting the preconception care intervention (PCCI) (week 0, 2, 4 and 6). Data are shown as means ± SEM. Per treatment, the same 8 mice were used at each time point

Mice fed the HF diet displayed impaired glucose tolerance compared with the CTRL_CTRL mice throughout the experiment (Fig. 4). More specifically, HF_HF mice showed significantly higher AUC (*P* < 0.05) and glucose peak (*P* < 0.05) concentrations compared with the CTRL_CTRL group at all time points. Despite the increased AUC for glucose in the HF_HF mice, the elimination rate (ER) was elevated after four (*P* < 0.1) and six (*P* < 0.05) weeks of PCCI compared with HF_CTRL and CTRL_CTRL mice (Supplementary Fig. 2).

The curves in Fig. 4 suggests an overall improvement in glucose tolerance in the HF_CTRL mice already after 2 weeks of PCCI. However only from 4 weeks of PCCI onwards, HF_CTRL values for AUC, ER and peak glucose concentrations were significantly decreased when compared with the HF_HF mice (*P* < 0.05) and even restored to the level of the control group.

Similarly, HF_CR mice showed a partial improved glucose tolerance after only 2 weeks of PCCI and a complete recovery after 4 weeks. However, feeding a calorie restricted diet for 6 weeks aggravated glucose tolerance characteristics again together with an upregulated ER, similar to the HF_HF mice.

Insulin tolerance tests (ITT) were performed on the same mice at all time points.

**Fig. 5:**
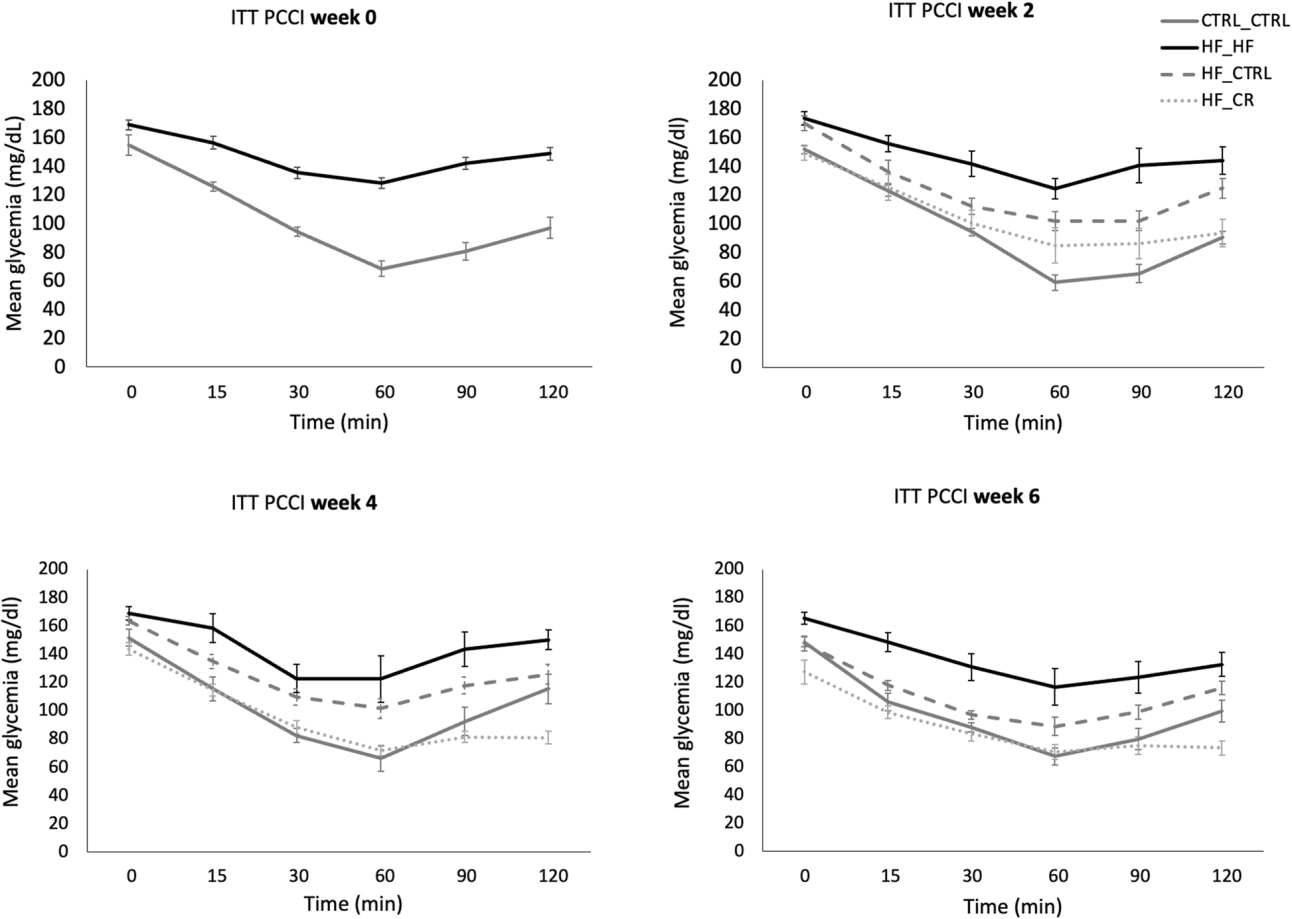
Serum glucose concentrations during an insulin sensitivity test (ITT) per treatment group at different time points after starting the preconception care intervention (PCCI) (week 0, 2, 4 and 6). Data are shown as means ± SEM from the same 8 mice per group per time point. Per treatment, the same 8 mice were used at each time point

HF_HF mice displayed an overall impaired insulin sensitivity compared with CTRL_CTRL mice throughout the experiment (Fig. 5). This was confirmed by a significantly higher AUC and a lower ER for glucose than the CTRL_CTRL group at all time points (Supplementary Fig. 3).

The curve in Fig. 5 showed a partially improved insulin sensitivity in HF_CTRL mice, illustrated by a steadily improving AUC for glucose while the ER was never statistically different from the HF group (Supplementary Fig. 3). However, the HF_CTRL mice never showed a completely recovered insulin sensitivity, even after 6 weeks of PCCI.

In contrast, a caloric restriction diet (HF_CR) was able to significantly improve insulin sensitivity after only 4 weeks of PCCI, substantiated by a significantly lower AUC. At 6 weeks of PCCI, overall insulin sensitivity of HF_CR mice was still restored, in contrast to the observed decreased glucose tolerance at this time point. However, ER for glucose did not improve when compared with the HF_HF mice at both time points as the HF_CR mice were not able to restore their glucose concentrations back to the basal levels within the tested timeframe of 120 min.

### Oocyte quality - fertility

#### Effect on oocyte recovery rate after hormonal stimulation

The average number of oocytes collected from the oviduct after hormonal stimulation was significantly lower in the HF_HF compared with the CTRL_CTRL group regardless of the PCCI time point (14 ± 5.5 vs. 19 ± 8.9; *P* < 0.05).

After 2 weeks of PCCI, no significant differences between the HF_HF and the PCCI groups were present. However, HF_CR mice yielded significantly more oocytes than both CTRL_CTRL and HF_HF mice after 4 weeks of PCCI (24 ± 10 vs. 15 ± 7; 24 ± 10 vs. 12 ± 5 respectively). This was not the case anymore after 6 weeks of PCCI. No other significant differences were present.

#### Mitochondrial ultrastructure in cumulus cells and oocytes

In the CTRL_CTRL oocytes, 5.25% (PCCI week 0) and 3.02% (PCCI week 6) of the evaluated mitochondria were categorized as structurally abnormal. Exposure to a HF diet (HF_HF group) significantly increased that percentage to 33.90% (*P* < 0.001) at PCCI week 0 which further increased to 46.44% at week 6 (Table 2). Both preconception care intervention groups displayed a significant improvement of mitochondrial ultrastructural abnormalities to only 9.95% in the HF_CTRL group and 9.56% in the HF_CR group compared with the HF_HF group. However, they were not completely restored as they still showed significantly higher percentage of mitochondrial abnormalities than CTRL_CTRL oocytes (9.95 and 9.56% versus 3.02%, respectively).

**Table 2:** Proportions of mitochondria with normal or abnormal ultrastructure (using TEM) in cumulus cells and oocytes from all treatment groups at PCCI week 0 and week 6

		Total mitochondria	Normal mitochondria	Abnormal mitochondria
**Oocytes**				
**PCCI week 0**	**HF_HF**	525	347 (66.10%)	178 (33.90%)^a^
	**CTRL_CTRL**	400	379 (94.75%)	21 (5.25%)^b^
**PCCI week 6**	**HF_HF**	562	301 (53,56%)	261 (46.44%)^a^
	**HF_CTRL**	442	398 (90.05%)	44 (9.95%)^b^
	**HF_CR**	586	530 (90.44%)	56 (9.56%)^b^
	**CTRL_CTRL**	497	482 (96.98%)	15 (3.02%)^c^
**Cumulus cells**				
**PCCI week 0**	**HF_HF**	305	301 (98.69%)	4 (1.31%)
	**CTRL_CTRL**	193	191 (98.96%)	2 (1.04%)
**PCCI week 6**	**HF_HF**	446	437 (97.98%)	9 (2.02%)
	**HF_CTRL**	318	312 (98.11%)	6 (1.89%)
	**HF_CR**	429	416 (96.97%)	13 (3.03%)
	**CTRL_CTRL**	504	497 (98.61%)	7 (1.39%)

Data are presented as proportions from total number of mitochondria evaluated, from 3 to 5 COCs per treatment group (from 3 to 5 mice per treatment group). Significant differences between the indicated treatment groups at the same PCCI time point are indicated with letters a, b and c (*P* < 0.05)

No significant differences in the ultrastructure of cumulus cell mitochondria could be observed at both time points (week 0 and 6 of PCCI) (Table 2).

#### Mitochondrial activity, localization of active mitochondria and ROS levels in oocytes

**Fig. 6:**
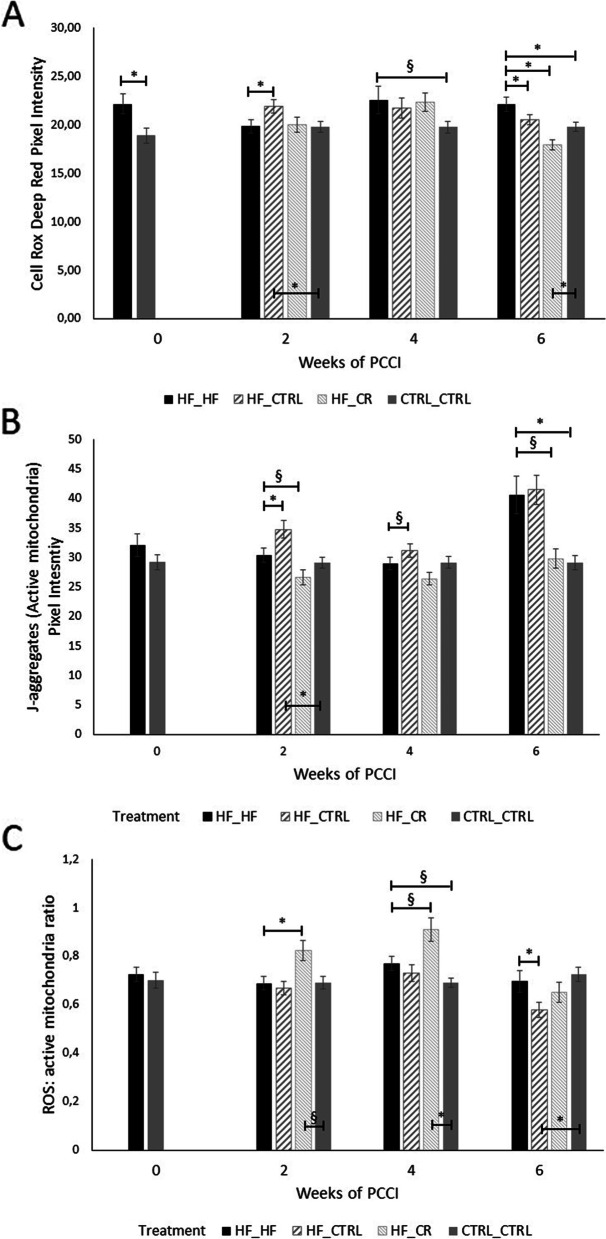
Quantification of ROS levels (**A**; CellRox Deep Red staining), mitochondrial activity (**B**; JC1- staining) and ROS: active mitochondria ratio (**C**) at different time points after starting the preconception care intervention (PCCI). Data are shown as means ± SEM from 1 to 3 oocytes per mouse and 6–8 mice per group per time point. Asterisks (*) indicate significant differences between indicated treatment groups within the same PCCI period (*P* < 0.05). Values labelled with “§” tend to be different from each other at 0.05 < *P* < 0.1

Oocytes collected from HF-fed mice displayed higher ROS levels compared with CTRL_CTRL oocytes at almost all time points (PCCI week 0 (*P* < 0.05), week 4 (*P* < 0.1) and week 6 (*P* < 0.05); Fig. 6A). However, when ROS levels were normalized for total number of active mitochondria, this effect disappeared (Fig. 6C). HF_HF oocytes only showed significantly higher mitochondrial activity than CTRL_CTRL oocytes at PCCI week 6 (*P* < 0.05; Fig. 6B).

HF_CTRL oocytes showed significantly higher ROS levels and mitochondrial activity than both reference groups after 2 weeks of PCCI (*P* < 0.05). These levels declined as the PCCI period continued. ROS levels were even significantly improved after 6 weeks of PCCI when compared with the HF_HF group (*P* < 0.05). This was associated with a significantly lower ratio of ROS: active mitochondria (*P* < 0.05).

In the same line as HF_CTRL mice, switching to a caloric restriction diet (HF_CR) significantly improved ROS levels only after 6 weeks of PCCI (*P* < 0.001), even lower than CTRL_CTRL oocytes (*P* < 0.05). In contrast to HF_CTRL, oocytes collected from HF_CR mice showed reduced mitochondrial activity after 2 and 6 weeks of PCCI when compared with the HF_HF group (*P* < 0.1). Interestingly, at 2 and 4 weeks, a significant increase was seen in the ROS levels when normalized for total number of active mitochondria. At PCCI week 6, this was not the case anymore.

**Fig. 7:**
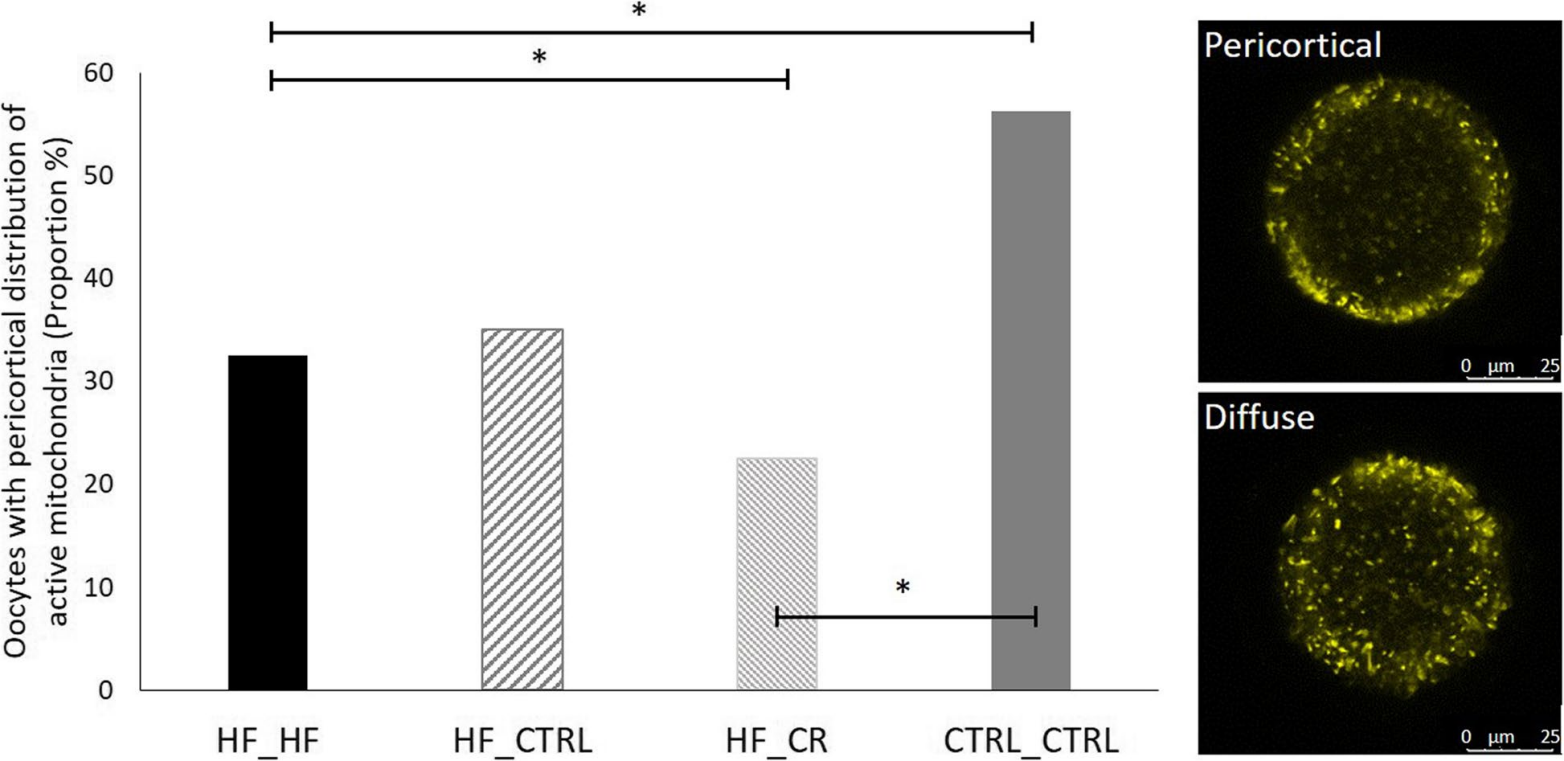
Percentage of oocytes with pericortical distribution of active mitochondria (diffuse/pericortical), pooled per treatment group regardless of time point. Data are shown from 32 to 43 oocytes (from 20 to 26 mice) per treatment group. Asterisks (*) indicate significant differences between indicated treatment groups (*P* < 0.05)

Since the number of oocytes used in this live cell confocal imaging was relatively low, data were pooled per treatment group and analyzed regardless of time points to assess pericortical localization of active mitochondria. The proportion of oocytes with pericortical distribution of active mitochondria was markedly lower in HF_HF-fed mice compared with CTRL_CTRL (*P* < 0.05 when using the merged data; Fig. 7). This was not improved in both PCCI groups and this condition was even worse in the HF_CR oocytes compared with the HF_HF oocytes. When dissecting these data for each time point, a similar trend was seen.

#### Oocyte mtDNA copy number

No significant differences were present between treatment groups after 2 weeks of PCCI. At PCCI week 4, oocytes from HF_HF mice displayed significantly higher mtDNA copy numbers than oocytes from the CTRL_CTRL group (*P* < 0.05; Supplementary Fig. 4). Oocytes collected from HF_CR mice showed a tendency to lower mtDNA copy numbers when compared with the HF_HF group (*P* < 0.1). At week 6 of the intervention, no differences between the treatment groups could be seen anymore.

#### Lipid droplet volume – Bodipy staining

**Fig. 8:**
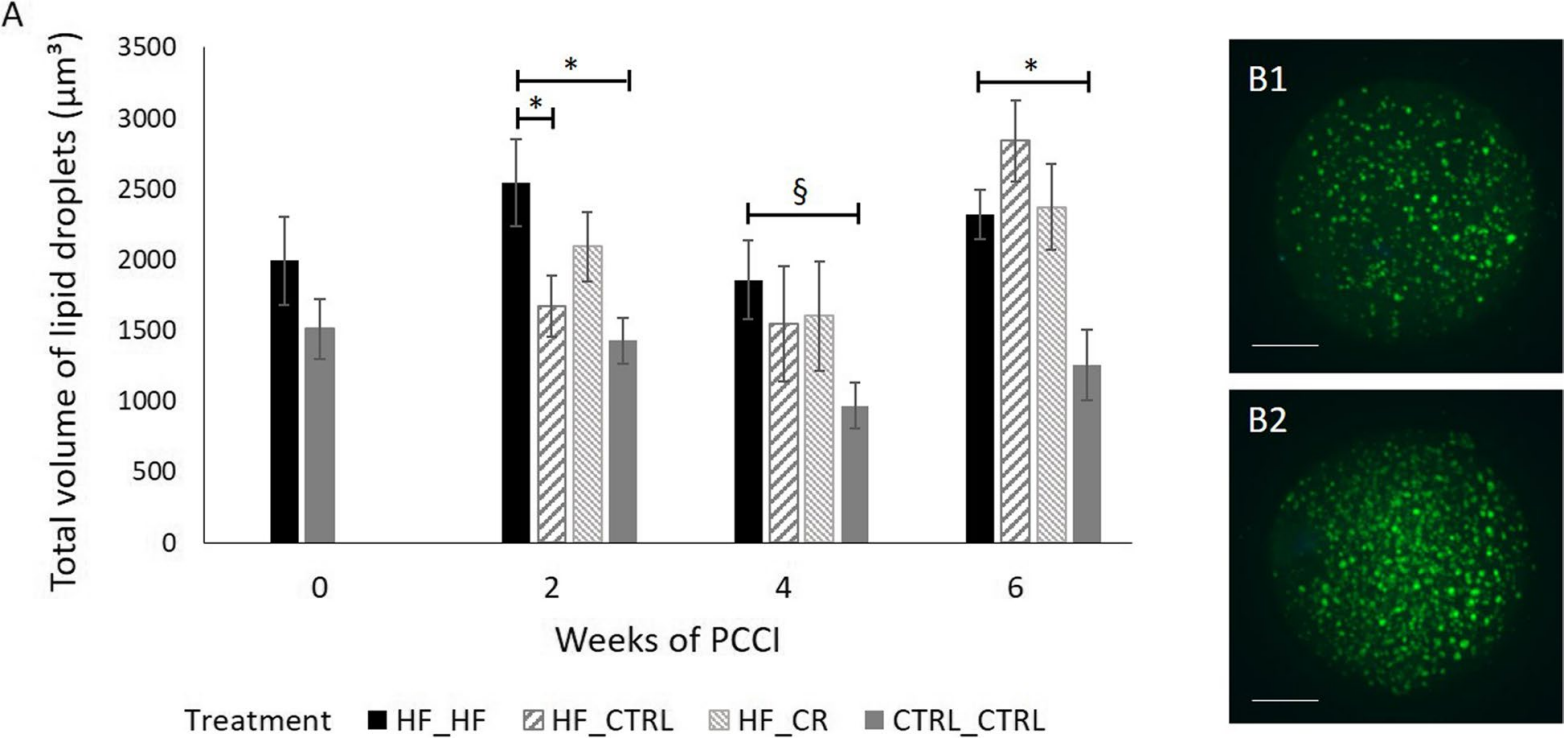
Representative confocal microscope images after BODIPY 493/503 staining showing lipid droplets (green, panel **B1** and **B2**) in oocytes collected from mice of all treatment groups, after 0, 2, 4 or 6 weeks of preconception care intervention (PCCI). Each image is a z-stack projection of 40 × 1 μm steps. Data are presented as means ± SEM from 1 to 3 oocytes per mouse, collected from 6 to 8 mice per group per time point (panel **A**). Asterisks (*) indicate significant differences between indicated treatment groups within the same PCCI period (*P* < 0.05). Values labelled with “§” tend to be different from each other at 0.05 < *P* < 0.1

Oocytes were examined for total lipid droplet volume using BODIPY 493/503 staining. Quantification of the z-stacks showed that oocytes collected from HF_HF mice displayed a higher lipid droplet volume than the CTRL_CTRL mice at PCCI week 2 (*P* < 0.05), 4 (*P* < 0.1) and 6 (*P* < 0.05) (Fig. 8a). Oocytes collected from HF_CTRL mice showed a significantly lower lipid droplet volume than mice on the high fat diet (HF_HF) after 2 weeks of PCCI (*P* < 0.05). However, at PCCI week 4, this was not the case anymore. Obese mice that switched to a caloric restriction diet (HF_CR) were not able to improve the elevated lipid content in the oocytes.

#### Pregnancy rates (after 6 weeks of PCCI)

Pregnancy rates (defined as proportion %) were significantly lower in the HF_HF mice compared with the control group (12.5% vs. 100%; 8 mice per treatment group). Interestingly, this was not the case anymore in mice that switched to a preconception diet intervention as they both showed significantly higher pregnancy rates than mice on the HF diet and similar rates when compared with the CTRL_CTRL group (87.5% in HF_CTRL mice and 100% in HF_CR mice).

It is important to keep in mind that pregnancy rates are based on a timed mating where male and female mice were housed together for four nights.

## Discussion

The aim of this study was to investigate whether dietary interventions of two, four or six weeks before conception were associated with an improved metabolic health, oocyte quality and fertility in an obese outbred mouse model. We confirmed that a HF diet exposure resulted in an obese phenotype characterized by a hampered metabolic health together with low oocyte quality and reduced fertility. When obese mice were switched from their obesogenic diet to a normal, control diet (HF_CTRL) for only 2 weeks, overall metabolic health (serum insulin and cholesterol, insulin sensitivity) was clearly improved. Extending the exposure period to 4 weeks further ameliorated their metabolic health as glucose tolerance was also significantly restored.

Submitting obese mice to a 30% calorie restricted diet also improved glucose tolerance and insulin sensitivity after 4 weeks of PCCI but other parameters measuring metabolic health (serum insulin, cholesterol and ALT) remained abnormal when compared with control mice. Submitting mice to this severely restricted feeding regime for 6 weeks negatively affected glucose tolerance.

The beneficial effects of both PCCI’s on oocyte quality were most prominent after 6 weeks as indicated by a reduction in ROS and mitochondrial ultrastructural abnormalities. At the end, pregnancy rates were significantly improved in both dietary interventions while oocyte lipid content was still abnormal after 6 weeks of PCCI.

### Metabolic health

We could confirm that HF diet feeding significantly increased body weight of Swiss outbred mice, resulting in an obese phenotype that was maintained throughout the whole experiment. In addition to the high body weight, obesity is also linked to reduced glucose tolerance, which we confirmed in this study [40]. The reported increased ER and glucose peak concentrations that were detected at PCCI week 4 and 6 can be linked to a renal loss of glucose due to the hyperglycemic state [41]. Next to that, the pancreas might have responded with a compensatory increased insulin release. To further confirm this hypothesis, insulin sensitivity of the mice was also tested. In addition to the significantly increased fasted serum insulin concentrations, HF_HF mice also displayed a significantly decreased insulin sensitivity at all time points when compared with the CTRL_CTRL group. These results clearly indicate that HF diet feeding to Swiss outbred mice resulted in a reduced insulin sensitivity, corresponding to a pre-diabetic state.

In addition to the reduced glucose tolerance and insulin sensitivity, HF_HF mice also showed hypercholesterolemia, as reported in many other studies on metabolic syndrome [18, 42]. Serum NEFA concentrations in this study were unchanged, as also reported by Podrini C*,* et al. [42] and Williams LM*,* et al. [43]. These observations should be interpreted with caution as animal handling and fasting may rapidly change NEFA concentrations, potentially masking important treatment effects. Interestingly, HF diet decreased serum triglyceride concentrations which has already been described in several murine studies [42, 44, 45]. Also in a human study, decreased circulating triglyceride concentrations were reported when 550 kcal of fat was added to the daily diet [46]. This decrease in circulating triglycerides can be caused by several possible mechanisms, like an inadequate export of triglycerides from the liver, a suppressed triglyceride production and/or an increased triglyceride clearance from the blood stream. In addition, the reduced serum triglyceride concentrations can also be linked to the elevated insulin concentrations observed in the HF_HF group since insulin can acutely inhibit hepatic VLDL secretion [47] and stimulates adipose triglyceride uptake via lipoprotein lipase [48]. In contrast, other studies report the opposite effect [49]. Clearly, additional research is needed to fully comprehend the mechanisms causing reduced serum triglyceride concentrations during HF diet feeding.

HF_CTRL mice already showed a partial normalization of the aberrant serum triglyceride and cholesterol profile after only 2 weeks of PCCI. This was paralleled with a clear loss in weight of 11%. Earlier research described a positive linear relationship between cholesterol and weight change [50]. Insulin sensitivity has also been reported to improve in proportion to the degree of weight loss [51]. While insulin serum concentrations were significantly improved, overall insulin sensitivity was only partially restored at PCCI week 2 as indicated by a decrease in glucose AUC which remained however significantly higher than CTRL_CTRL mice. Both fasting blood glucose concentrations and glucose tolerance were not significantly improved after 2 weeks of PCCI. These data indicate that switching from a HF to an ad libitum control diet for only a short time frame of 2 weeks already shows some positive effects on metabolic health with regards to weight, serum lipid profile and insulin sensitivity. However, a complete recovery of the glucose tolerance was only present after 4 weeks of diet normalization, coinciding with 14% of weight loss. This is in line with the results of Reynolds KA*,* et al. [20], who also reported a complete recovery of glucose tolerance in mice that underwent diet normalization, however, these analyses were only performed at eight weeks of PCCI. Although fasting insulin serum concentrations were decreased in the HF_CTRL group at all timepoints, there was no complete recovery of overall insulin sensitivity, even after 6 weeks of PCCI.

Metabolic health was also assessed in HF_CR mice. Switching from a high fat to a 30% caloric restriction diet (HF_CR) also resulted in a reduction in weight which however continued during the whole PCCI period, ranging from 17% weight loss at PCCI week 2 to 27% weight loss at week 6. In contrast to HF_CTRL mice, HF_CR mice showed a lack of improved cholesterol concentrations at all time points. Earlier research in obese women associated major weight loss with a late rise in serum cholesterol, possibly from mobilization of adipose cholesterol stores. However, the increased cholesterol resolved when weight loss stabilized [52].

Furthermore, serum collected from HF_CR mice contained significantly higher ALT concentrations than both reference groups at all time points. This has been seen in other studies [53–56]. The rapid mobilization of intra- and extrahepatic fat stores may represent a hepatotoxic factor, explaining the leakage of this liver enzyme in the bloodstream. Such damage of the hepatocytes can, furthermore, cause an inadequate export of triglycerides from the liver which explains the low serum triglyceride concentrations seen in the HF_CR mice at all time points.

With regards to glucose tolerance and insulin sensitivity HF_CR mice showed a very similar response as HF_CTRL mice. After 2 weeks of PCCI, no significant improvements in glucose tolerance and only a partial improvement in insulin sensitivity were present.

After 4 weeks on a calorie restricted diet, the glucose tolerance was completely restored. Furthermore, and in contrast to HF_CTRL mice, HF_CR mice completely restored their insulin sensitivity to the level of the CTRL_CTRL mice. However, calorie restricted mice were not able to recover from the hypoglycemia induced by the insulin injection by the end of the insulin tolerance test (Time 120′ on the graph (Fig. 5)). As suggested above, liver health is reduced in this HF_CR group (high ALT concentrations), the capacity to restore from this hypoglycemia may be jeopardized due to reduced rates of liver gluconeogenesis and glycogenolysis. Furthermore, the prolonged calorie restricted diet can lead to glycogen depletion in muscle and liver [57].

In addition, maintaining HF_CR mice for 6 weeks on this restricted diet showed to be detrimental for overall glucose tolerance, accompanied by a higher peak glucose concentration and a faster clearance. Most probably, a higher insulin independent glucose uptake will play an important role in this. Furthermore, this phenomenon can also be explained by a state of pseudo-diabetes, as firstly reported by Claude Bernard in 1848 [58]. Although a very low calorie diet has been accepted as a powerful treatment to improve health and to reverse type 2 diabetes in humans, some reports describe the development of a diabetes-like state [59–61]. However, the disease profile of pseudo-diabetes is characterized by 5 major symptoms. Of these symptoms, in our experimental set-up, only glucose intolerance and hyperinsulinemia support the hypothesis of a pseudo-diabetes state in the HF_CR mice as they did show normoglycemia and recovered insulin sensitivity after 6 weeks of PCCI.

Taken together, although serum insulin and cholesterol concentrations of HF_CTRL mice already showed a promising improvement after 2 weeks of PCCI, this time period is too short to completely restore glucose tolerance and insulin sensitivity in both HF_CTRL and HF_CR mice. The most promising metabolic improvements were present in HF_CTRL mice (including a restored glucose tolerance) at PCCI week 4, as HF_CR mice still showed an aberrant serum profile. A longer period of intervention did not further improve metabolic health in the HF_CTRL group and was even detrimental in HF_CR mice.

### Oocyte quality

Our results confirm that oocytes collected from HF_HF mice contained a significantly higher lipid droplet volume than oocytes from CTRL_CTRL mice [18]. Lipids are an important source for energy production in the oocyte [62]. However, obesity leads to hyperlipidemia, resulting in lipid accumulation and storage in cells other than adipocytes. This ultimately results in lipotoxicity as the intracellular accumulation of lipids causes damage to cellular organelles like mitochondria, resulting in increased ROS and oxidative stress [63, 64]. Whether the observed increase in oocyte intracellular lipids is due to increased active uptake from the follicular microenvironment or due to a shift in energy pathways in the oocyte as a result of the different nutrient availability, needs further study.

Oocytes collected from HF_HF mice also exhibited higher ROS levels than the CTRL_CTRL group at almost all time points, as shown by Cell Rox Deep Red staining pixel intensity [18]. Elevated intracellular ROS concentrations and thus increased oxidative stress in oocytes from obese individuals results in mitochondrial dysfunction and reduced oocyte quality [16, 18]. Igosheva N*,* et al. [17] linked increased ROS production in oocytes from obese female mice to a higher mtDNA copy number. A high mtDNA copy number in oocytes has been linked with a higher risk of implantation failure in human embryos [65]. Oocytes collected from HF_HF mice contained higher ROS and significantly higher mtDNA copy number than oocytes from CTRL_CTRL oocytes after 11 weeks (at week 4 of the PCCI period) on their respective diet. We suggest that this increase in mtDNA copy number can be linked to a defective mitophagy and/or increased mitochondrial biogenesis during oogenesis to compensate for the increasing number of defective mitochondria [17, 66, 67]. In contrast, at PCCI week 6, a reduction in mtDNA copy numbers was present in oocytes from HF_HF mice. This might indicate that the earlier compensatory feedback that resulted in increased mitochondrial biogenesis is no longer present. At the same timepoint we confirmed a very high proportion of mitochondrial ultrastructural abnormalities [18]. Although not reported in oocytes yet, persistent nutrient surplus, like e.g. HF diet exposure, has been shown to override the adaptation and can lead to mitochondrial overloading and dysfunction in muscles [68].

The mitochondrial dysfunction in oocytes caused by obesity also manifests at several other levels, ranging from an increased mitochondrial activity (JC-1 pixel intensity) to an improper mitochondrial distribution during final maturation, as confirmed in the present study [17, 18]. Increased mitochondrial membrane potential has been linked with reduced oocyte developmental competence in both mice and humans [69]. In addition, HF_HF mature oocytes displayed a significantly lower percentage of active mitochondria that were pericortically localized when compared with CTRL_CTRL oocytes. Van Blerkom J*,* et al. [70] reported that in the mature oocyte, high polarized mitochondria normally occupy a circumferential domain immediately subjacent to the plasma membrane, also described as ‘vanguard mitochondrial polarity’ [71]. This pericortical localization supports ATP levels during embryo cleavage [72], is required for successful sperm penetration and cortical granule exocytosis [73]. As HF_HF oocytes clearly show a lower percentage of oocytes containing pericortically localized active mitochondria, this might result in decreased fertilization and cleavage rates.

Furthermore, in this study, pregnancy rates of HF_HF mice were also significantly lower, as reported by Skaznik-Wikiel ME*,* et al. [74].

Focusing on the HF_CTRL mice, ovulated oocytes exhibited a lower lipid droplet content after only 2 weeks of PCCI. This was correlated with the reported decrease in blood total cholesterol levels, again suggesting similar biochemical changes in the ovarian follicular fluid. As mitochondrial activity was increased, this suggests an increased use of lipids for fatty-acid β-oxidation to produce energy for the oocyte. Dunning KR*,* et al. [75] illustrated that upregulation of *β*-oxidation increased oocyte developmental competence. The increased mitochondrial activity can also be linked to a possible improved responsiveness of the cumulus cells to glucose after 2 weeks of diet normalization. This results in increased pyruvate uptake in the oocyte, leading to an increased oxidative phosphorylation [76]. In this study, the increased mitochondrial activity also coincided with an increase in ROS, possibly indicating the presence of oxidative stress.

As the PCCI continued beyond the timeframe of folliculogenesis (i.e. 3 weeks) (PCCI week 4 and 6), there was a clear transition in HF_CTRL oocytes towards (again) a high lipid droplet volume, accompanied by high mitochondrial activity but a significant reduction in ROS levels and ratio of ROS: active mitochondria. As glucose and pyruvate, besides lipids, are critical substrates for successful oocyte and embryo development (reviewed by [76]), we suggest that the HF_CTRL oocyte relies on the carbohydrate metabolism of the cumulus cells and uses its end products as the major energy source, resulting in an increase of intracellular lipids. The ability to efficiently store fatty acids in lipid droplets might decrease lipotoxic effects and can be a method to store energy for the preimplantation development [77–79]. These observations may suggest that oocytes from HF_CTRL mice are more metabolically efficient and have a higher antioxidant capacity.

Above mentioned outcome parameters indicate a possibly improved oocyte quality. However, the overall percentage of oocytes with pericortically localized active mitochondria did not improve in HF_CTRL oocytes and was even similar to the percentage reported in HF_HF oocytes.

To summarize, we see a clear shift in oocytes from HF_CTRL mice towards the use of different energy pathways around the timeframe that folliculogenesis (i.e. 3 weeks) did no longer took place in a high fat environment. This might indicate that the preconception care intervention should be followed for a period longer than what is needed for folliculogenesis. The timeframe of 6 weeks PCCI showed to be the most optimal.

On the other hand, oocytes collected from the HF_CR group still exhibited a high volume of lipid droplets at each PCCI time point. Weight loss has been linked to increased lipid mobilization from the adipose tissue, resulting in increased NEFA concentrations in the blood stream that were reflected in the FF [26, 80]. These increased FF NEFA concentrations might lead to increased fatty acid uptake in the oocyte, resulting in a high lipid droplet volume in the oocyte. In addition to the high lipid content, mean ratio of ROS: active mitochondria was even higher than the HF_HF oocytes after 2 and 4 weeks of PCCI. This indicates an upregulation of mitochondrial β-oxidation due to the high lipid content, resulting in excessive ROS production leading to oxidative stress. However, at PCCI week 6, a reduction in total ROS levels and ratio of ROS: active mitochondria was present in HF_CR oocytes, indicating a decrease in oxidative stress.

To our knowledge, nothing is known regarding the effect of a CR diet on mitochondrial activity in oocytes. However, earlier research in other tissues indicated that a restricted calorie intake or an increased physical activity can significantly improve mitochondrial integrity and function and protect against metabolic syndrome [68, 81, 82]. Similar to the HF_CTRL mice, overall percentage of oocytes with pericortically localized active mitochondria did not improve in HF_CR mice and was even lower than HF_HF mice. However, after 6 weeks of PCCI, HF_CR oocytes showed lower mitochondrial activity than HF_HF oocytes. This indicates that switching to a CR diet for 6 weeks might result in oocytes with an improved mitochondrial function.

As an additional endpoint parameter, oocyte mitochondrial ultrastructural abnormalities were categorized. Oocytes from both HF_CTRL and HF_CR mice showed a significantly reduced proportion of mitochondrial ultrastructural abnormalities at PCCI week 6 compared with HF_HF mice. Along with the other assessed oocyte quality parameters, these data suggest that exposure to a preconception diet intervention for 6 weeks seems to result in the ovulation of a better quality oocyte that was able to develop in healthier conditions as the complete process of folliculogenesis took place in a non-high fat environment. However, the mitochondrial ultrastructural abnormalities were not fully restored to the level of the control group.

Folliculogenesis lasts 3–4 months in humans and only 3 weeks in mice [83]. During this process, the growing follicle and the enclosed oocyte are very sensitive to changes in its micro-environment which impacts the quality of the oocyte at the moment of ovulation [84, 85]. This emphasizes the importance of the different PCCI period lengths that were selected in the present study. Seeing oocyte quality significantly improving after 4 and 6 weeks of PCCI suggests that the dormant primordial follicle pool is not really affected by the HF dietary insult or that oocyte recovery mechanisms during folliculogenesis are able to repair. However, any remnant negative impact on oocyte quality at PCCI week 4 and 6 may, to some extent, contradict this. Furthermore, the not fully recovered metabolic health of course may still exert negative effects during the growth and maturation of the follicle. From this, it should be clear that more in depth research is needed to better understand these concepts.

As a final validation of our findings regarding oocyte quality, pregnancy rates were assessed at PCCI week 6. Both HF_CTRL and HF_CR mice showed restored pregnancy rates, hereby indicating that switching from a HF to an ad libitum control or 30% CR diet had a positive effect on overall fertility.

Taken together, a PCCI period of 6 weeks shows to be the most promising in both PCCI groups. Oocytes from HF_CTRL mice that stayed on the control diet for 6 weeks seemed to switch towards the use of different energy pathways, which may suggest a more metabolically efficient phenotype with a higher antioxidant capacity. At the end of the PCCI period, oocytes from HF_CR mice displayed reduced ROS levels and restored mitochondrial activity. Additional endpoint parameters assessed at week 6 indicated that mitochondrial ultrastructural abnormalities were drastically lower in both PCCI groups compared with the HF_HF group and also pregnancy rates were restored.

## Limitations and extrapolation potential to the human situation

As mentioned before, human studies are often confronted with a lot of possible confounders (high drop-out rates, ethnical and social background,…), leading to a lack of scientifically substantiated guidelines. Therefore, using a mouse model for this research has several advantages and provides us with fundamental information, including the specific impact on oocyte quality. Mice are small, fertile and have a short gestational period. However, in contrast to humans, mice are poly-ovulatory animals and translating the results from this mouse study to the human setting should be done with caution. However, the use of an outbred mouse strain, the specific implementation of PCCI lengths related to the duration of mouse folliculogenesis (i.e. 3 weeks) together with an in depth study of the weight changes and the metabolic health effects, should help in understanding the potential consequences of our study’s fundamental insights for a human fertility setting. Furthermore, ongoing research in our laboratory is providing deeper insights in potential postnatal health effects of described preconception care interventions.

## Conclusions

This research is an important step in providing fundamental insights regarding the impact of diet normalization or a calorie restricted diet for two, four or six weeks as preconception care intervention strategies on metabolic health and oocyte quality in HF-diet fed obese outbred mice.

We confirmed that obesity has a detrimental effect on both metabolic health and oocyte quality in a HF diet-fed outbred mouse model.

Based on the collected data, switching to an ad libitum control diet (HF_CTRL) for a time frame of 4 weeks with a weight loss of 14% is a good approach to improve metabolic health. Oocyte quality parameters assessed already showed some promising improvements at PCCI week 4. However, these were further ameliorated at PCCI week 6.

Switching to a 30% caloric restriction diet (HF_CR) for 6 weeks showed to be a too extreme intervention, due to its negative impact on glucose tolerance, insulin concentrations and liver health and due to the lack of improved serum lipid concentrations. In contrast, oocyte quality parameters in the HF_CR mice only showed a significantly improved quality after 6 weeks of PCCI as indicated by a restored mitochondrial activity, reduction in ROS and mitochondrial ultrastructural abnormalities.

## Supplementary Information

**Additional file 1 **: **Supplementary Figure 1.** Classification of different forms of mitochondrial ultrastructure. Mitochondria were considered normal when spherical (a) or spherical with regular vacuoles (b). Mitochondrial abnormalities include vacuolation with loose inner membrane structures (c), electron dense foci (d), dumbbell shapes with vacuolation (e), dumbbell shapes (f), rose petal appearance (g) or degeneration (h). **Supplementary Figure 2.** Peak glucose concentration, area under the curve (AUC) and elimination rate (ER) of the glucose tolerance test of all treatment groups at different time points after starting the preconception care intervention (PCCI) (week 0, 2, 4 and 6).  **Supplementary Figure 3.** Glucose area under the curve (AUC) and elimination rate (ER) of the insulin tolerance test **(**ITT) of all treatment groups at different time points after starting the preconception care intervention (PCCI).  **Supplementary Figure 4.** mtDNA copy numbers of all treatment groups at different time points after starting the preconception care intervention (PCCI).

## Data Availability

All data generated or analyzed during this study are included in this published article and its supplementary information files.

## Abbreviations

ALT: Alanine aminotransferase
AUC: Area under the curve
COC: Cumulus oocyte complex
CR: Caloric restriction
CTRL: Control
eCG: Equine chorionic gonadotropin
ER: Elimination rate
FF: Follicular fluid
GTT: Glucose tolerance test
hCG: Human chorionic gonadotropin
HF: High fat
ITT: Insulin tolerance test
IVF: In vitro fertilization
mtDNA: Mitochondrial DNA
NEFA: Non-esterified fatty acids
PCCI: Preconception care intervention
ROS: Reactive oxygen species
TEM: Transmission electron microscopy

## References

[1] Eckel RH, Alberti KG, Grundy SM, Zimmet PZ (2010). The metabolic syndrome. Lancet.

[2] Engin A (2017). The definition and prevalence of obesity and metabolic syndrome. Adv Exp Med Biol.

[3] WHO (2018). Facts about overweight and obesity.

[4] Bluher M (2019). Obesity: global epidemiology and pathogenesis. Nat Rev Endocrinol.

[5] Dahlhoff M, Pfister S, Blutke A, Rozman J, Klingenspor M, Deutsch MJ, Rathkolb B, Fink B, Gimpfl M, Hrabe de Angelis M (2014). Peri-conceptional obesogenic exposure induces sex-specific programming of disease susceptibilities in adult mouse offspring. Biochim Biophys Acta.

[6] Jungheim ES, Moley KH (2010). Current knowledge of obesity's effects in the pre- and periconceptional periods and avenues for future research. Am J Obstet Gynecol.

[7] Samuelsson AM, Matthews PA, Argenton M, Christie MR, McConnell JM, Jansen EH, Piersma AH, Ozanne SE, Twinn DF, Remacle C (2008). Diet-induced obesity in female mice leads to offspring hyperphagia, adiposity, hypertension, and insulin resistance: a novel murine model of developmental programming. Hypertension.

[8] Klenov VE, Jungheim ES (2014). Obesity and reproductive function: a review of the evidence. Curr Opin Obstet Gynecol.

[9] Metwally M, Cutting R, Tipton A, Skull J, Ledger WL, Li TC (2007). Effect of increased body mass index on oocyte and embryo quality in IVF patients. Reprod BioMed Online.

[10] Zain MM, Norman RJ (2008). Impact of obesity on female fertility and fertility treatment. Womens Health (Lond).

[11] Robker RL (2008). Evidence that obesity alters the quality of oocytes and embryos. Pathophysiology.

[12] Sutton-McDowall ML, Gilchrist RB, Thompson JG (2010). The pivotal role of glucose metabolism in determining oocyte developmental competence. Reproduction.

[13] Valckx SD, Arias-Alvarez M, De Pauw I, Fievez V, Vlaeminck B, Fransen E, Bols PE, Leroy JL (2014). Fatty acid composition of the follicular fluid of normal weight, overweight and obese women undergoing assisted reproductive treatment: a descriptive cross-sectional study. Reprod Biol Endocrinol.

[14] Robker RL, Wu LL, Yang X (2011). Inflammatory pathways linking obesity and ovarian dysfunction. J Reprod Immunol.

[15] Snider AP, Wood JR (2019). Obesity induces ovarian inflammation and reduces oocyte quality. Reproduction.

[16] Grindler NM, Moley KH (2013). Maternal obesity, infertility and mitochondrial dysfunction: potential mechanisms emerging from mouse model systems. Mol Hum Reprod.

[17] Igosheva N, Abramov AY, Poston L, Eckert JJ, Fleming TP, Duchen MR, McConnell J (2010). Maternal diet-induced obesity alters mitochondrial activity and redox status in mouse oocytes and zygotes. PLoS One.

[18] Marei WFA, Smits A, Mohey-Elsaeed O, Pintelon I, Ginneberge D, Bols PEJ, Moerloose K, Leroy J (2020). Differential effects of high fat diet-induced obesity on oocyte mitochondrial functions in inbred and outbred mice. Sci Rep.

[19] Wu LL, Dunning KR, Yang X, Russell DL, Lane M, Norman RJ, Robker RL (2010). High-fat diet causes lipotoxicity responses in cumulus-oocyte complexes and decreased fertilization rates. Endocrinology.

[20] Reynolds KA, Boudoures AL, Chi MM, Wang Q, Moley KH (2015). Adverse effects of obesity and/or high-fat diet on oocyte quality and metabolism are not reversible with resumption of regular diet in mice. Reprod Fertil Dev.

[21] Lassi ZS, Dean SV, Mallick D, Bhutta ZA (2014). Preconception care: delivery strategies and packages for care. Reprod Health.

[22] Pasquali R (2006). Obesity, fat distribution and infertility. Maturitas.

[23] Jensen MD, Ryan DH, Apovian CM, Ard JD, Comuzzie AG, Donato KA, Hu FB, Hubbard VS, Jakicic JM, Kushner RF (2013). AHA/ACC/TOS guideline for the management of overweight and obesity in adults: a report of the American College of Cardiology/American Heart Association Task Force on Practice Guidelines and The Obesity Society. J Am Coll Cardiol.

[24] Aksungar FB, Sarikaya M, Coskun A, Serteser M, Unsal I (2017). Comparison of intermittent fasting versus caloric restriction in obese subjects: a two year follow-up. J Nutr Health Aging.

[25] Andersen CJ, Fernandez ML (2013). Dietary strategies to reduce metabolic syndrome. Rev Endocr Metab Disord.

[26] Chusyd DE, Wang D, Huffman DM, Nagy TR (2016). Relationships between rodent white adipose fat pads and human white adipose fat depots. Front Nutr.

[27] Marei WFA, Van Raemdonck G, Baggerman G, Bols PEJ, Leroy J (2019). Proteomic changes in oocytes after in vitro maturation in lipotoxic conditions are different from those in cumulus cells. Sci Rep.

[28] Sim KA, Partridge SR, Sainsbury A (2014). Does weight loss in overweight or obese women improve fertility treatment outcomes? A systematic review. Obes Rev.

[29] Einarsson S, Bergh C, Friberg B, Pinborg A, Klajnbard A, Karlstrom PO, Kluge L, Larsson I, Loft A, Mikkelsen-Englund AL (2017). Weight reduction intervention for obese infertile women prior to IVF: a randomized controlled trial. Hum Reprod.

[30] Mutsaerts MA, van Oers AM, Groen H, Burggraaff JM, Kuchenbecker WK, Perquin DA, Koks CA, van Golde R, Kaaijk EM, Schierbeek JM (2016). Randomized trial of a lifestyle program in obese infertile women. N Engl J Med.

[31] Tsagareli V, Noakes M, Norman RJ (2006). Effect of a very-low-calorie diet on in vitro fertilization outcomes. Fertil Steril.

[32] Norman RJ, Mol BWJ (2018). Successful weight loss interventions before in vitro fertilization: fat chance?. Fertil Steril.

[33] Lan L, Harrison CL, Misso M, Hill B, Teede HJ, Mol BW, Moran LJ (2017). Systematic review and meta-analysis of the impact of preconception lifestyle interventions on fertility, obstetric, fetal, anthropometric and metabolic outcomes in men and women. Hum Reprod.

[34] Pruessner JC, Kirschbaum C, Meinlschmid G, Hellhammer DH (2003). Two formulas for computation of the area under the curve represent measures of total hormone concentration versus time-dependent change. Psychoneuroendocrinology.

[35] Rezende LF, Santos GJ, Santos-Silva JC, Carneiro EM, Boschero AC (2012). Ciliary neurotrophic factor (CNTF) protects non-obese Swiss mice against type 2 diabetes by increasing beta cell mass and reducing insulin clearance. Diabetologia.

[36] Komatsu K, Iwase A, Mawatari M, Wang J, Yamashita M, Kikkawa F (2014). Mitochondrial membrane potential in 2-cell stage embryos correlates with the success of preimplantation development. Reproduction.

[37] De Biasi S, Gibellini L, Cossarizza A (2015). Uncompensated polychromatic analysis of mitochondrial membrane potential using JC-1 and multilaser excitation. Curr Protoc Cytom.

[38] Wakefield SL, Lane M, Schulz SJ, Hebart ML, Thompson JG, Mitchell M (2008). Maternal supply of omega-3 polyunsaturated fatty acids alter mechanisms involved in oocyte and early embryo development in the mouse. Am J Physiol Endocrinol Metab.

[39] Livak KJ, Schmittgen TD (2001). Analysis of relative gene expression data using real-time quantitative PCR and the 2(−Delta Delta C(T)) method. Methods.

[40] Buettner R, Scholmerich J, Bollheimer LC (2007). High-fat diets: modeling the metabolic disorders of human obesity in rodents. Obesity (Silver Spring).

[41] Liman MNP, Jialal I (2021). Physiology, glycosuria.

[42] Podrini C, Cambridge EL, Lelliott CJ, Carragher DM, Estabel J, Gerdin AK, Karp NA, Scudamore CL, Sanger Mouse Genetics P, Ramirez-Solis R, White JK (2013). High-fat feeding rapidly induces obesity and lipid derangements in C57BL/6N mice. Mamm Genome.

[43] Williams LM, Campbell FM, Drew JE, Koch C, Hoggard N, Rees WD, Kamolrat T, Thi Ngo H, Steffensen IL, Gray SR, Tups A (2014). The development of diet-induced obesity and glucose intolerance in C57BL/6 mice on a high-fat diet consists of distinct phases. PLoS One.

[44] Biddinger SB, Almind K, Miyazaki M, Kokkotou E, Ntambi JM, Kahn CR (2005). Effects of diet and genetic background on sterol regulatory element-binding protein-1c, stearoyl-CoA desaturase 1, and the development of the metabolic syndrome. Diabetes.

[45] Guo J, Jou W, Gavrilova O, Hall KD (2009). Persistent diet-induced obesity in male C57BL/6 mice resulting from temporary obesigenic diets. PLoS One.

[46] Meugnier E, Bossu C, Oliel M, Jeanne S, Michaut A, Sothier M, Brozek J, Rome S, Laville M, Vidal H (2007). Changes in gene expression in skeletal muscle in response to fat overfeeding in lean men. Obesity (Silver Spring).

[47] Lewis GF, Uffelman KD, Szeto LW, Weller B, Steiner G (1995). Interaction between free fatty acids and insulin in the acute control of very low density lipoprotein production in humans. J Clin Invest.

[48] Kessler JI (1963). Effect of diabetes and insulin on the activity of myocardial and adipose tissue lipoprotein lipase of rats. J Clin Invest.

[49] Ginsberg HN, Zhang YL, Hernandez-Ono A (2005). Regulation of plasma triglycerides in insulin resistance and diabetes. Arch Med Res.

[50] Poobalan A, Aucott L, Smith WC, Avenell A, Jung R, Broom J, Grant AM (2004). Effects of weight loss in overweight/obese individuals and long-term lipid outcomes--a systematic review. Obes Rev.

[51] Ferrannini E, Camastra S (1998). Relationship between impaired glucose tolerance, non-insulin-dependent diabetes mellitus and obesity. Eur J Clin Investig.

[52] Phinney SD, Tang AB, Waggoner CR, Tezanos-Pinto RG, Davis PA (1991). The transient hypercholesterolemia of major weight loss. Am J Clin Nutr.

[53] Friis R, Vaziri ND, Akbarpour F, Afrasiabi A (1987). Effect of rapid weight loss with supplemented fasting on liver tests. J Clin Gastroenterol.

[54] Gasteyger C, Larsen TM, Vercruysse F, Astrup A (2008). Effect of a dietary-induced weight loss on liver enzymes in obese subjects. Am J Clin Nutr.

[55] Hoy MK, Heshka S, Allison DB, Grasset E, Blank R, Abiri M, Heymsfield SB (1994). Reduced risk of liver-function-test abnormalities and new gallstone formation with weight loss on 3350-kJ (800-kcal) formula diets. Am J Clin Nutr.

[56] Kreitzman SN, Pedersen M, Budell W, Nichols D, Krissman P, Clements M (1984). Safety and effectiveness of weight reduction using a very-low-calorie formulated food. Arch Intern Med.

[57] Xu S, Chen G, Chunrui L, Liu C (2015). The preventive and therapeutic effect of caloric restriction therapy on type 2 diabetes mellitus, treatment of type 2 diabetes.

[58] Lundbaek K (1948). Metabolic abnormalities in starvation diabetes. Yale J Biol Med.

[59] Blagosklonny MV (2019). Fasting and rapamycin: diabetes versus benevolent glucose intolerance. Cell Death Dis.

[60] Fontana L, Klein S, Holloszy JO (2010). Effects of long-term calorie restriction and endurance exercise on glucose tolerance, insulin action, and adipokine production. Age (Dordr).

[61] Koffler M, Kisch ES (1996). Starvation diet and very-low-calorie diets may induce insulin resistance and overt diabetes mellitus. J Diabetes Complicat.

[62] Sutton-McDowall ML, Feil D, Robker RL, Thompson JG, Dunning KR (2012). Utilization of endogenous fatty acid stores for energy production in bovine preimplantation embryos. Theriogenology.

[63] Suzuki M (2017). Regulation of lipid metabolism via a connection between the endoplasmic reticulum and lipid droplets. Anat Sci Int.

[64] Wu LL, Norman RJ, Robker RL (2011). The impact of obesity on oocytes: evidence for lipotoxicity mechanisms. Reprod Fertil Dev.

[65] Fragouli E, McCaffrey C, Ravichandran K, Spath K, Grifo JA, Munne S, Wells D (2017). Clinical implications of mitochondrial DNA quantification on pregnancy outcomes: a blinded prospective non-selection study. Hum Reprod.

[66] Barrientos A, Casademont J, Cardellach F, Ardite E, Estivill X, Urbano-Marquez A, Fernandez-Checa JC, Nunes V (1997). Qualitative and quantitative changes in skeletal muscle mtDNA and expression of mitochondrial-encoded genes in the human aging process. Biochem Mol Med.

[67] Luzzo KM, Wang Q, Purcell SH, Chi M, Jimenez PT, Grindler N, Schedl T, Moley KH (2012). High fat diet induced developmental defects in the mouse: oocyte meiotic aneuploidy and fetal growth retardation/brain defects. PLoS One.

[68] Cheng Z, Almeida FA (2014). Mitochondrial alteration in type 2 diabetes and obesity: an epigenetic link. Cell Cycle.

[69] Acton BM, Jurisicova A, Jurisica I, Casper RF (2004). Alterations in mitochondrial membrane potential during preimplantation stages of mouse and human embryo development. Mol Hum Reprod.

[70] Van Blerkom J, Davis P, Mathwig V, Alexander S (2002). Domains of high-polarized and low-polarized mitochondria may occur in mouse and human oocytes and early embryos. Hum Reprod.

[71] Van Blerkom J, Davis P (2006). High-polarized (Delta Psi m(HIGH)) mitochondria are spatially polarized in human oocytes and early embryos in stable subplasmalemmal domains: developmental significance and the concept of vanguard mitochondria. Reprod BioMed Online.

[72] Van Blerkom J, Davis P, Alexander S (2003). Inner mitochondrial membrane potential (DeltaPsim), cytoplasmic ATP content and free Ca2+ levels in metaphase II mouse oocytes. Hum Reprod.

[73] Van Blerkom J, Davis P (2007). Mitochondrial signaling and fertilization. Mol Hum Reprod.

[74] Skaznik-Wikiel ME, Swindle DC, Allshouse AA, Polotsky AJ, McManaman JL (2016). High-fat diet causes subfertility and compromised ovarian function independent of obesity in mice. Biol Reprod.

[75] Dunning KR, Cashman K, Russell DL, Thompson JG, Norman RJ, Robker RL (2010). Beta-oxidation is essential for mouse oocyte developmental competence and early embryo development. Biol Reprod.

[76] Richani D, Dunning KR, Thompson JG, Gilchrist RB (2021). Metabolic co-dependence of the oocyte and cumulus cells: essential role in determining oocyte developmental competence. Hum Reprod Update.

[77] Ferguson EM, Leese HJ (2006). A potential role for triglyceride as an energy source during bovine oocyte maturation and early embryo development. Mol Reprod Dev.

[78] Hillman N, Flynn TJ (1980). The metabolism of exogenous fatty acids by preimplantation mouse embryos developing in vitro. J Embryol Exp Morphol.

[79] Sturmey RG, Reis A, Leese HJ, McEvoy TG (2009). Role of fatty acids in energy provision during oocyte maturation and early embryo development. Reprod Domest Anim.

[80] Valckx SD, De Pauw I, De Neubourg D, Inion I, Berth M, Fransen E, Bols PE, Leroy JL (2012). BMI-related metabolic composition of the follicular fluid of women undergoing assisted reproductive treatment and the consequences for oocyte and embryo quality. Hum Reprod.

[81] Martin-Montalvo A, de Cabo R (2013). Mitochondrial metabolic reprogramming induced by calorie restriction. Antioxid Redox Signal.

[82] Toledo FG, Goodpaster BH (2013). The role of weight loss and exercise in correcting skeletal muscle mitochondrial abnormalities in obesity, diabetes and aging. Mol Cell Endocrinol.

[83] Clarke H (2017). Control of mammalian oocyte development by interactions with the maternal follicular environment. Results Probl Cell Differ.

[84] Best D, Bhattacharya S (2015). Obesity and fertility. Horm Mol Biol Clin Investig.

[85] Valckx SD, Van Hoeck V, Arias-Alvarez M, Maillo V, Lopez-Cardona AP, Gutierrez-Adan A, Berth M, Cortvrindt R, Bols PE, Leroy JL (2014). Elevated non-esterified fatty acid concentrations during in vitro murine follicle growth alter follicular physiology and reduce oocyte developmental competence. Fertil Steril.

