# Supplementary material for: Diet normalization or caloric restriction as a preconception care strategy to improve metabolic health and oocyte quality in obese outbred mice
Source: Reprod Biol Endocrinol. 2021 Nov 4;19:166. doi: 10.1186/s12958-021-00848-4 (PMC8567997; doi:10.1186/s12958-021-00848-4)
Supplement: Supplementary file 1 — Additional file 1 : Supplementary Figure 1. Classification of different forms of mitochondrial ultrastructure. Mitochondria were considered normal when spherical (a) or spherical with regular vacuoles (b). Mitochondrial abnormalities include vacuolation with loose inner membrane structures (c), electron dense foci (d), dumbbell shapes with vacuolation (e), dumbbell shapes (f), rose petal appearance (g) or degeneration (h). Supplementary Figure 2. Peak glucose concentration, area under the curve (AUC) and elimination rate (ER) of the glucose tolerance test of all treatment groups at different time points after starting the preconception care intervention (PCCI) (week 0, 2, 4 and 6). Supplementary Figure 3. Glucose area under the curve (AUC) and elimination rate (ER) of the insulin tolerance test (ITT) of all treatment groups at different time points after starting the preconception care intervention (PCCI). Supplementary Figure 4. mtDNA copy numbers of all treatment groups at different time points after starting the preconception care intervention (PCCI). [file 12958_2021_848_MOESM1_ESM.docx]

**Diet normalization or caloric restriction as a preconception care strategy to improve metabolic health and oocyte quality in obese outbred mice.**

Anouk Smits^1,^*, Waleed FA Marei^1^, Diane De Neubourg ^2^, Jo LMR Leroy^1^

^1^Gamete Research Centre, Laboratory for Veterinary Physiology and Biochemistry, Department of Veterinary Sciences, University of Antwerp, 2610 Wilrijk, Belgium

^2^Centre for Reproductive Medicine - Antwerp University Hospital, University of Antwerp, Wilrijkstraat 10, 2650 Edegem, Belgium

*Corresponding author

Supplementary information

Supplementary figure 1


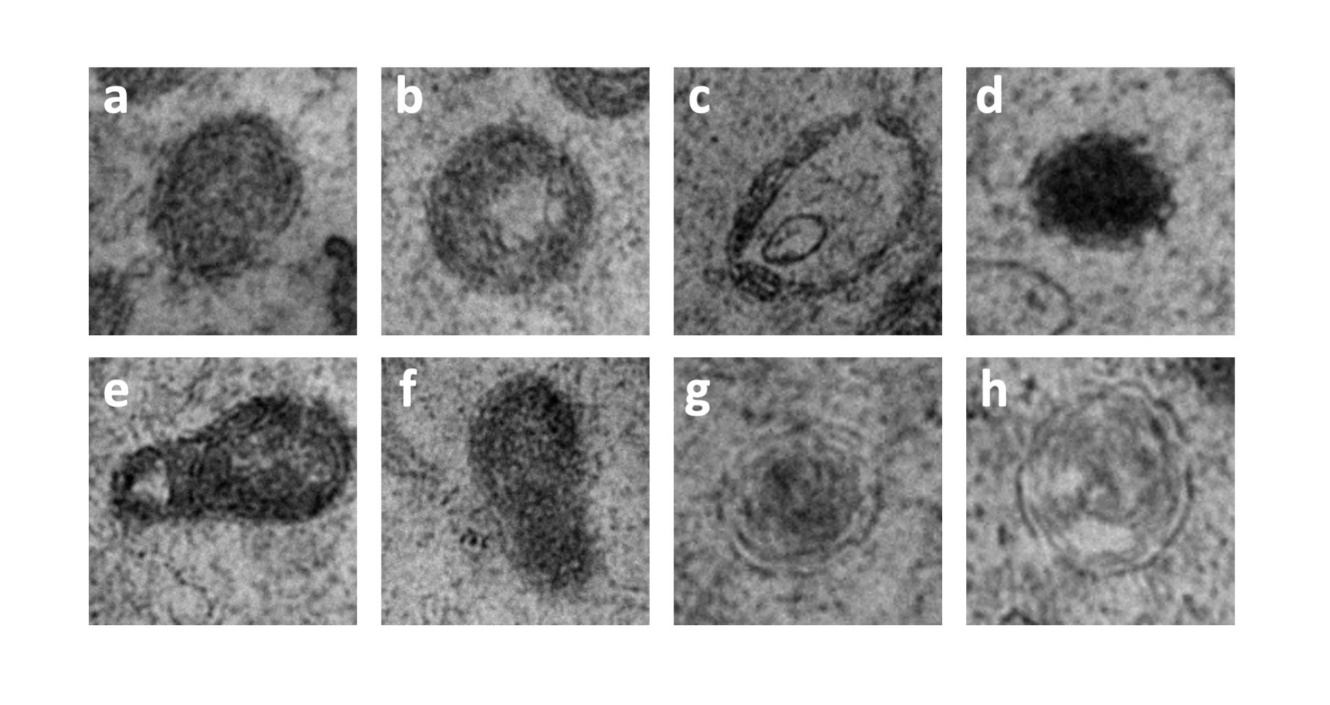
**Supplementary Fig. 1**  **Classification of different forms of mitochondrial ultrastructure**. Mitochondria were considered normal when spherical (a) or spherical with regular vacuoles (b). Mitochondrial abnormalities include vacuolation with loose inner membrane structures (c), electron dense foci (d), dumbbell shapes with vacuolation (e), dumbbell shapes (f), rose petal appearance (g) or degeneration (h).

Supplementary figure 2


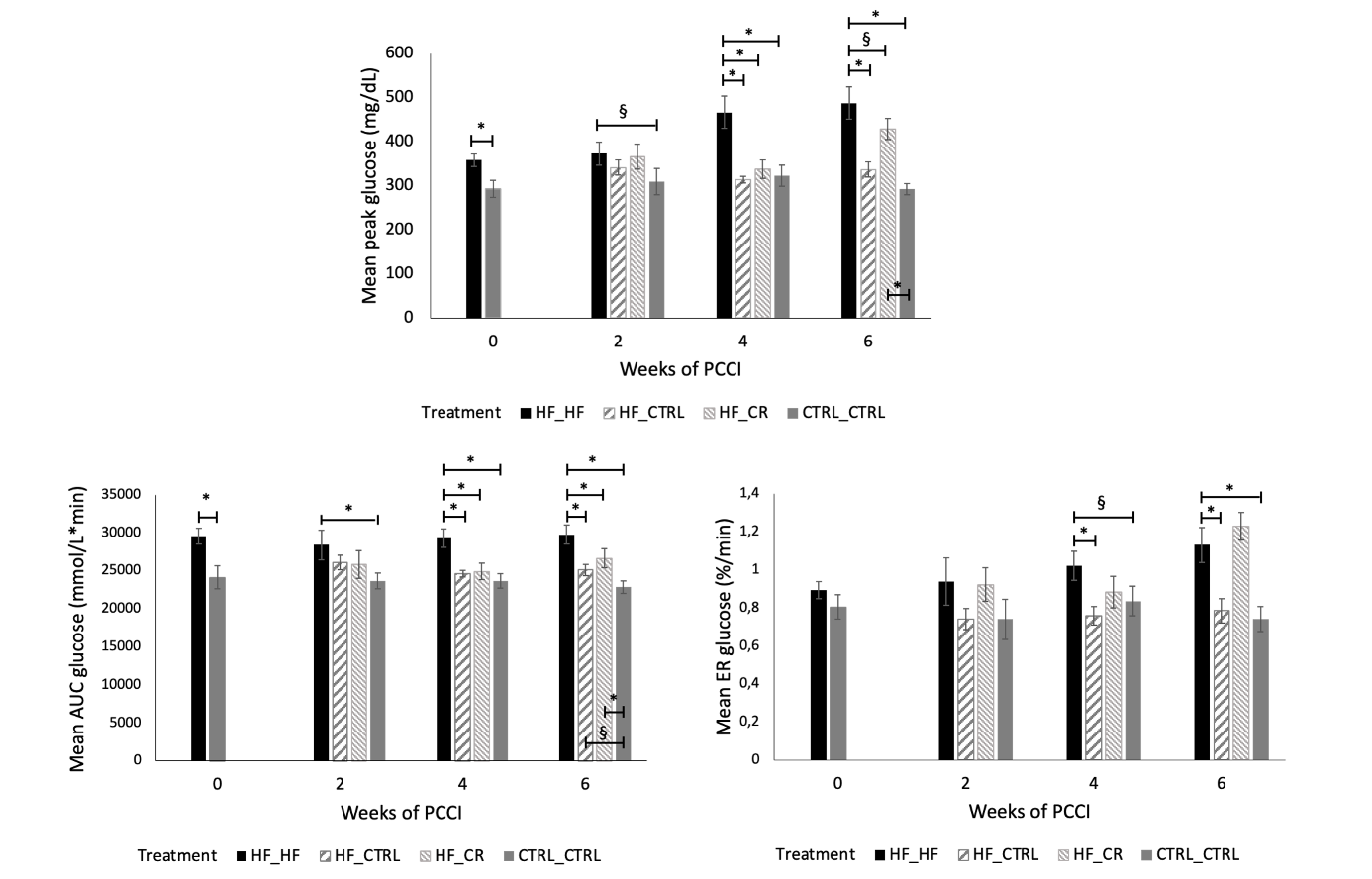


**Supplementary Fig. 2**  **Peak glucose concentration, area under the curve (AUC) and elimination rate (ER)** of the **glucose tolerance test** of all treatment groups at different time points after starting the preconception care intervention (PCCI) (week 0, 2, 4 and 6). Data are shown as means ± SEM from 8 mice per group per time point. Asterisks (*) indicate significant differences between the indicated treatment groups within the same PCCI period (*P*<0.05). Values labelled with “§” tend to be different from each other at 0.05 < *P* <0.1.

Supplementary figure 3


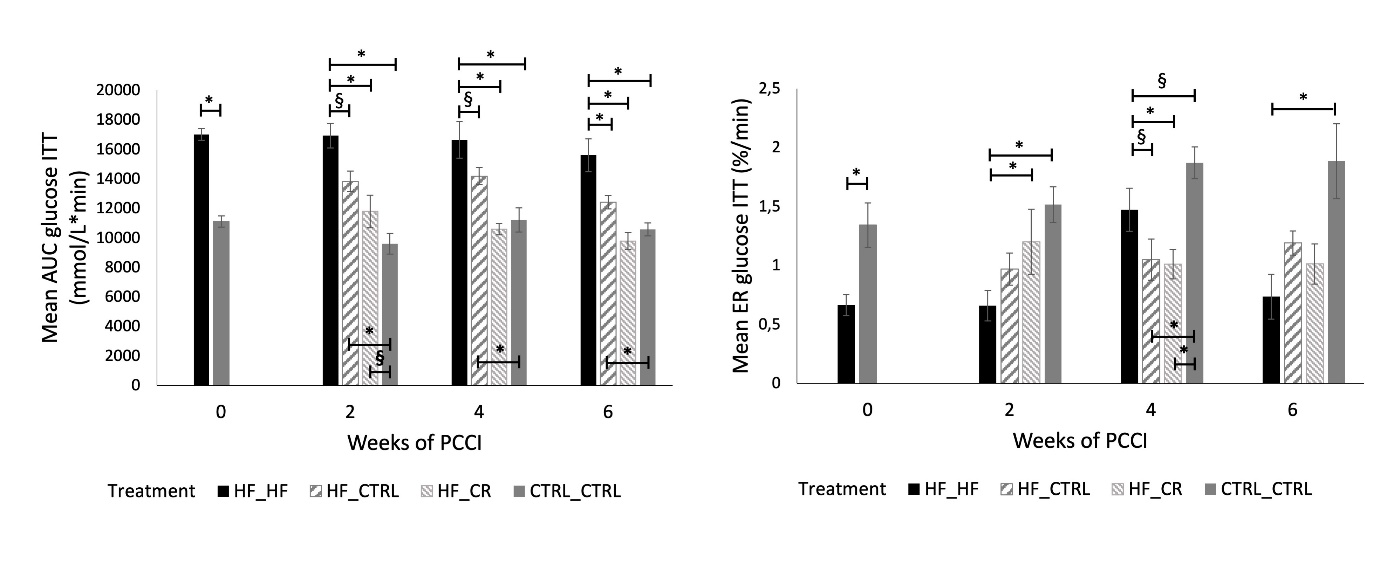
**Supplementary Fig. 3**  Glucose **area under the curve (AUC) and elimination rate (ER)** of the **insulin tolerance test** (ITT) of all treatment groups at different time points after starting the preconception care intervention (PCCI). Data are shown as means ± SEM from 8 mice per group per time point. Asterisks (*) indicate significant differences between indicated treatment groups within the same PCCI period (*P*<0.05). Values labelled with “§” tend to be different from each other at 0.05 < *P* <0.1.

Supplementary figure 4


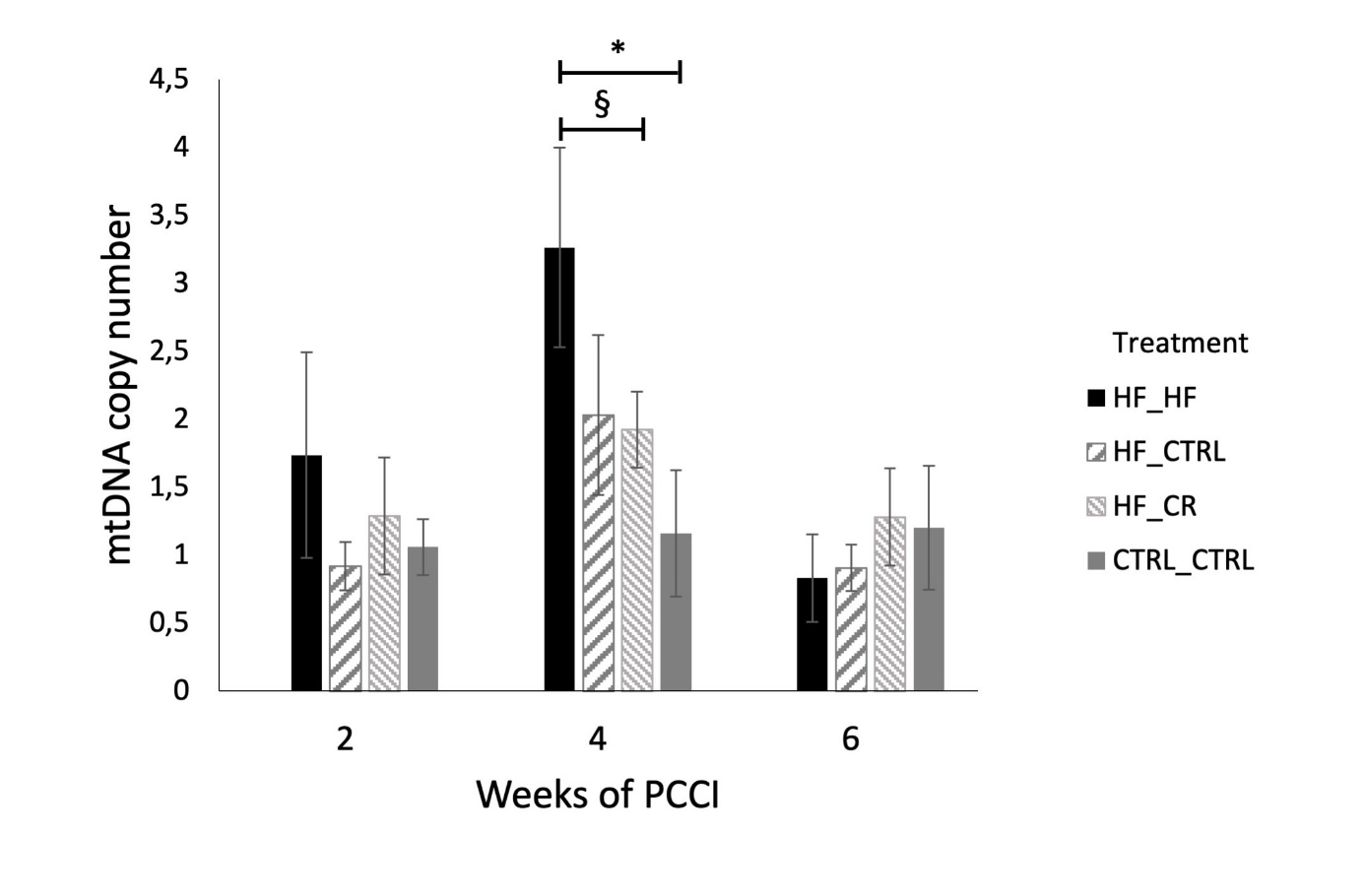
**Supplementary Fig. 4**  **mtDNA copy numbers** of all treatment groups at different time points after starting the preconception care intervention (PCCI). Data are shown as means ± SEM from pools of oocytes (11 ≤ n ≥ 42) per treatment group collected from 3-5 mice per group per time point. Asterisks (*) indicate significant differences between indicated treatment groups within the same PCCI period (*P*<0.05). Values labelled with “§” tend to be different from each other at 0.05 < *P* <0.1.
